# Do all mice smell the same? Chemosensory cues from inbred and wild mouse strains elicit stereotypic sensory representations in the accessory olfactory bulb

**DOI:** 10.1186/s12915-021-01064-7

**Published:** 2021-06-28

**Authors:** Rohini Bansal, Maximilian Nagel, Romana Stopkova, Yizhak Sofer, Tali Kimchi, Pavel Stopka, Marc Spehr, Yoram Ben-Shaul

**Affiliations:** 1 Department of Medical Neurobiology, Institute for Medical Research Israel Canada, Faculty of Medicine, The Hebrew University of Jerusalem, Jerusalem, Israel; 2 Department of Chemosensation, Institute for Biology II, RWTH Aachen University, Aachen, Germany; 3 BIOCEV group, Department of Zoology, Faculty of Science, Charles University, Prague, Czech Republic; 4 Department of Neurobiology, Weizmann Institute of Science, Rehovot, Israel

**Keywords:** Vomeronasal system, Accessory olfactory bulb, Wild mouse stimuli, Innate responses, Stimulus representations

## Abstract

**Background:**

For many animals, chemosensory cues are vital for social and defensive interactions and are primarily detected and processed by the vomeronasal system (VNS). These cues are often inherently associated with ethological meaning, leading to stereotyped behaviors. Thus, one would expect consistent representation of these stimuli across different individuals. However, individuals may express different arrays of vomeronasal sensory receptors and may vary in the pattern of connections between those receptors and projection neurons in the accessory olfactory bulb (AOB). In the first part of this study, we address the ability of individuals to form consistent representations despite these potential sources of variability. The second part of our study is motivated by the fact that the majority of research on VNS physiology involves the use of stimuli derived from inbred animals. Yet, it is unclear whether neuronal representations of inbred-derived stimuli are similar to those of more ethologically relevant wild-derived stimuli.

**Results:**

First, we compared sensory representations to inbred, wild-derived, and wild urine stimuli in the AOBs of males from two distinct inbred strains, using them as proxies for individuals. We found a remarkable similarity in stimulus representations across the two strains. Next, we compared AOB neuronal responses to inbred, wild-derived, and wild stimuli, again using male inbred mice as subjects. Employing various measures of neuronal activity, we show that wild-derived and wild stimuli elicit responses that are broadly similar to those from inbred stimuli: they are not considerably stronger or weaker, they show similar levels of sexual dimorphism, and when examining population-level activity, cluster with inbred mouse stimuli.

**Conclusions:**

Despite strain-specific differences and apparently random connectivity, the AOB can maintain stereotypic sensory representations for broad stimulus categories, providing a substrate for common stereotypical behaviors. In addition, despite many generations of inbreeding, AOB representations capture the key ethological features (i.e., species and sex) of wild-derived and wild counterparts. Beyond these broad similarities, representations of stimuli from wild mice are nevertheless distinct from those elicited by inbred mouse stimuli, suggesting that laboratory inbreeding has indeed resulted in marked modifications of urinary secretions.

**Supplementary Information:**

The online version contains supplementary material available at 10.1186/s12915-021-01064-7.

## Background

As organisms interact with their environment, their brain generates sensory representations of the objects in it. These representations, often a product of both innate hardwired elements and learning, amount to a mapping from external stimulus space to internal neuronal space. It is assumed that stimuli that are perceived as similar also generate similar neuronal representations, and thus, when different individuals agree on which stimuli are similar, they share similar representations of those stimuli.

Here, we address this topic in the context of chemosensory signaling. In mice, as in many other animals, chemosensation is a key mode of communication. Unlike neutral cues, whose valence and significance often depend on individual experience, cues from other organisms are often innately endowed with ethological significance [1, 2]. For example, a male mouse will likely avoid a predator, approach a receptive female mouse, and exhibit antagonistic behavior towards another male individual. In many vertebrates including mice, a dedicated chemosensory system, the vomeronasal system, processes cues from other organisms. The sensory organ of the VNS is the vomeronasal organ, which contains vomeronasal sensory neurons that project their axons to the AOB. The principal AOB neurons, mitral/tufted cells (MTCs), largely project to limbic regions including the vomeronasal amygdala [3, 4].

Yet, while consistent responses to chemical stimuli require a stereotypical representation of chemical space, it is not clear to what extent such stereotypy is present in the first central processing stage of the VNS, the AOB. Notably, receptor repertoires have been shown to change significantly across strains (and presumably among individual wild mice) due to strain-specific differences in genetics, expression patterns, and experiential factors [5–8]. Furthermore, in the VNS, glomerular connectivity between sensory neurons and AOB MTCs does not obey an obvious order as it so clearly does in the main olfactory system [9–13], but see also [13]. Although the existence of as yet undiscovered organizational rules within the AOB cannot be ruled out, and although labeled line circuits within the VNS have been found [14], at the cellular level, the AOB seems dramatically less organized than the main olfactory bulb [9], providing further incentive to test whether representations of chemical stimulus space are similar across mice.

**Fig. 1.**
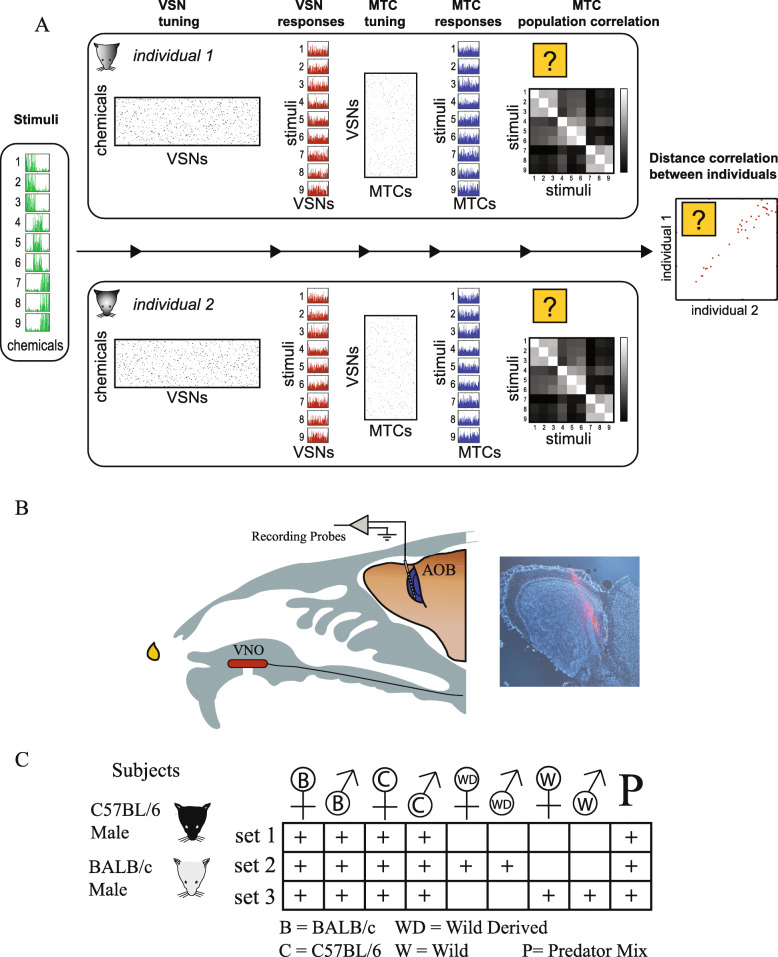
Experiment design. **a** The conceptual framework for our analyses. The figure describes key stages in the processing of complex stimuli by the VNS. In this manuscript, we focus on the distance between representations of distinct stimuli as illustrated by the correlation matrices and on the distance between the representations across different strains of mice. Both aspects are illustrated by the question marks. See the “Introduction” section for a detailed explanation of this panel. **b** Schematic of the experimental preparation for recording AOB responses. The inset shows an image of a DiI-colored electrode tract in the external cell layer of the AOB. **c** Stimulus name legend and stimuli used in each of the three datasets in the manuscript. The + signs indicate stimuli that were included in each data set. Male and female symbols indicate the sex of the stimulus donor, and the letters within them indicate the strain, as defined at the bottom of the table. Subject icons indicate the strain of the mouse from which recordings were made. These stimulus and subject symbols are used in other figures throughout the manuscript

The framework for our analysis is illustrated in Fig. 1a. The left panel shows nine hypothetical complex stimuli, defined by the levels of distinct molecular species (green bars). In this example, there are three groups of stimuli. Members of each group share similar levels of molecular content. Stimuli are detected by two different individuals (upper and lower rectangles). Any similarity need not depend on an absolute neuronal metric, but rather on the relative distance among stimuli, in neuronal coding space. The neuronal representation is generated in two stages. First, the stimuli elicit a certain peripheral response by activating an array of vomeronasal sensory neurons (VSNs) in each individual (red bars). This VSN response is determined by (***i***) the sensitivity of each VSN receptor type to each of the stimulus molecules and (***ii***) the levels of such molecules in each stimulus. At the next stage, receptor activation patterns are read by arrays of MTCs, each of which samples inputs from a number of glomeruli, resulting in a certain response pattern (blue bars). It is these MTC response patterns that constitute the raw data for our analyses. In our experiments, we record from random subsets of these MTCs, and based on those, we calculate (***i***) the similarity of stimulus representations in each individual (correlation matrices in Fig. 1a) and (***ii***) the similarity of representations *between* the two individuals (scatter plot in Fig. 1a).

Practically (Fig. 1b, c), we compared stimulus-induced AOB activity patterns in adult males from two distinct mouse strains, BALB/c (BC) and C57BL/6 (C57). AOB activity was measured using multisite electrode arrays, after stimulus application to the nostril and electrical activation of the vomeronasal organ (see the “Methods” section and [15]). The two inbred strains that we use here as subjects are commonly used in chemosensory research and are clearly distinct in terms of their lineage [16], which is likely to affect both their secretions and the receptor array [8, 17–24]. Leveraging the genetic homogeneity within each group, we treated these groups as “clones” of individuals, allowing us to assess the degree to which these “individuals” maintain similar representations of stimulus space.

To enhance the ethological validity of our analysis, we included in our stimulus sets not only urine from inbred mice, but also from both wild-derived and wild animals. As we show below, there exists a high degree of similarity across the two inbred subject strains in neuronal representations of ethologically relevant stimulus sets. This corresponds to the first interpretation of the manuscript’s title, referring to *smelling* in the active sense.

The second interpretation of the title question refers to the odors *emitted by* mice. Presently, virtually all experiments on VNS physiology employ urinary stimuli from inbred mice. While the use of inbred secretions facilitates stimulus collection and allows standardization across studies, it is not clear to what extent inbred stimuli are representative of more ethologically relevant wild-derived stimuli. Laboratory inbreeding could have modified the nature of chemical secretions, and consequently, their perception by other mice. Indeed, it has been shown that the chemical composition of wild mouse urine is different from that of inbred mouse urine [18]. This is significant not only from an evolutionary, but also from a practical perspective: if there are marked qualitative and/or quantitative differences in composition, and hence in the neuronal representations of wild as compared to inbred derived stimuli, one might question the conclusions based on a large body of work using inbred secretions. To address this issue, we analyzed responses to male and female mouse urine from both wild-derived [25] and wild mice [26] and compared these to responses elicited by urine from the two commonly used inbred strains (BALB/c and C57BL/6). Referring to the scheme outlined in Fig. 1a, this approach involves comparing whether stimuli from inbred and wild mice are represented similarly at the AOB level. Using various measures of neuronal activity, including population-level metrics (e.g., using correlation matrices as in Fig. 1a), we find that wild-derived and wild mouse stimuli do, in fact, elicit responses that are broadly similar to those from inbred stimuli. This indicates that despite many generations of inbreeding, the AOB has the capacity to encode the key ethological features of wild-derived and wild counterparts. Yet, importantly, beyond these broad similarities, we show that AOB level representations of stimuli from wild mice are noticeably distinct from representations of inbred mouse stimuli, indicating that laboratory inbreeding has indeed resulted in potentially meaningful modifications of urinary secretions.

## Results

**Fig. 2.**
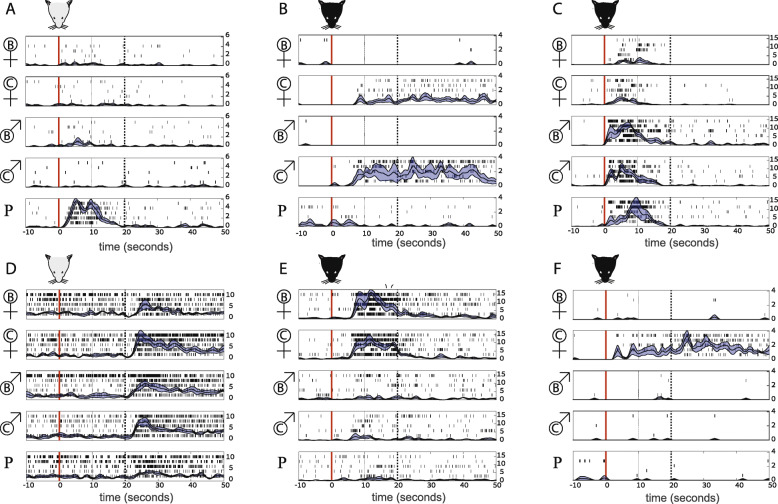
Data examples. Examples of responses of 6 single units to the five stimuli in stimulus set 1. The examples illustrate responses following application (red horizontal line) and stimulation (black-dotted line). Values to the right denote firing rates in Hz. The two panels on the left show neurons recorded in BC males. The other neurons were recorded in C57 males

We recorded AOB responses after controlled stimulus delivery to the VNO (Fig. 1b) in adult BC and C57 male mice. Several sets of stimuli were used (Fig. 1c), all of which included five basic stimuli: male and female urine from the C57 and BC strains and predator urine. In our experiments, we interleave the presentation of each of the stimuli in each dataset, in a pseudorandom order, generating at least 4 (and usually 5) repeated measurements for each stimulus. For each stimulus, we first apply a 2 μl drop of urine to the nostril. This step is denoted as *application* and is shown as a red vertical line in the examples of Fig. 2. Following a 20 s delay, we electrically stimulate the sympathetic nerve trunk, with the goal of inducing VNO suction (see the “Methods” section for details). This step is denoted as *stimulation* and is shown by the broken vertical black lines in Fig. 2. The goal of the electrical stimulation is to activate the VNO pump (e.g., Fig. 2d). In some experiments, however, we routinely observe responses prior to electrical stimulation (e.g., Fig. 2a). In the present set of experiments, the fraction of such responses was unusually high and we therefore quantified responses as mean firing rate changes (compared to the pre-stimulus baseline) following the entire 50 s epoch after stimulus *application*. This expanded temporal window captures neuronal activity induced following both application-induced and stimulation-induced VNO suction.

**Fig. 3.**
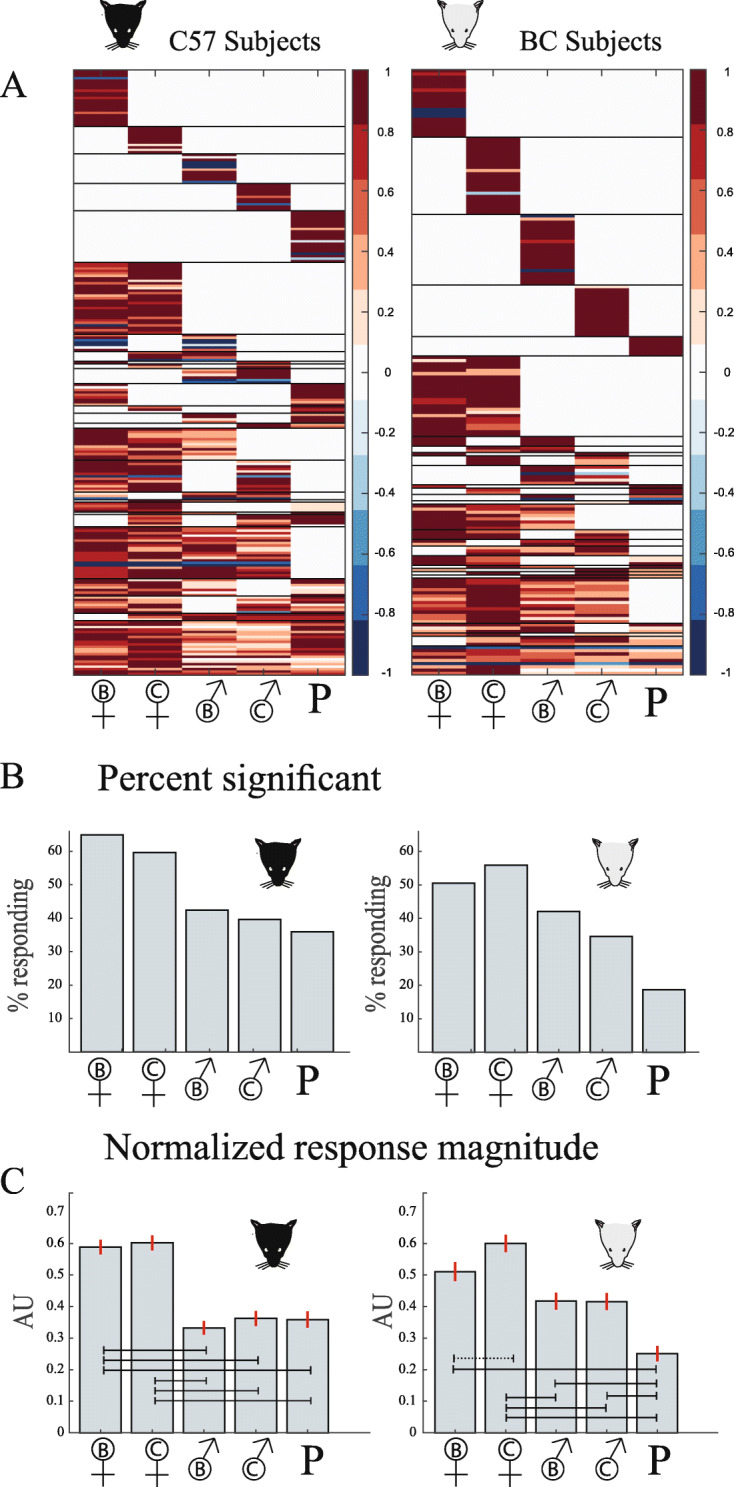
Basic features of the dataset. **a** Normalized response matrices. In these matrices, the mean response of each neuron (depicted in one row) is normalized to a value between −1 and 1. Each column corresponds to one stimulus. Non-significant responses are set to 0. Each matrix corresponds to all units recorded in a single strain. Raw and normalized matrices with significant responses are shown in Additional File 1. **b** Percent of neurons with significant responses to each of the stimuli. **c** Normalized response magnitude. In this representation, each neuron’s responses are normalized as in panel **a**. This normalization ensures that all neurons have the same contribution to the population response magnitude. Vertical error bars represent standard errors of the mean. Horizontal lines represent significant differences among stimulus pairs using a one-way non-parametric ANOVA (Kruskal-Wallis test). Broken lines indicate *p* values below 0.05, while solid lines indicate values below 0.01. See Additional File 2 for specific *p* values for this and all other pairwise comparisons

To visualize responses across the entire set of responsive neurons, we show in Fig. 3a the normalized response matrices of neurons with significant responses (C57, *N* = 245, 20 sessions from 14 mice, BC, *N* = 188, 20 sessions from 14 mice). Raw response matrices are shown in Additional File 1. Although not identical, response matrices from the two strains share several common features. Two basic features are the fraction of neurons with significant responses to each of the stimuli (Fig. 3b) and the mean (normalized) responses of the population to each of the stimuli (Fig. 3c, Additional File 2). Despite some variation among these two measures across the two subject strains, it is clear that (in the male mice tested here) female stimuli elicit more intense responses than either male or predator stimuli, an observation consistent with previous studies by others and us [27–29].

### Population-level response distances are similar in the AOBs of the two strains

**Fig. 4.**
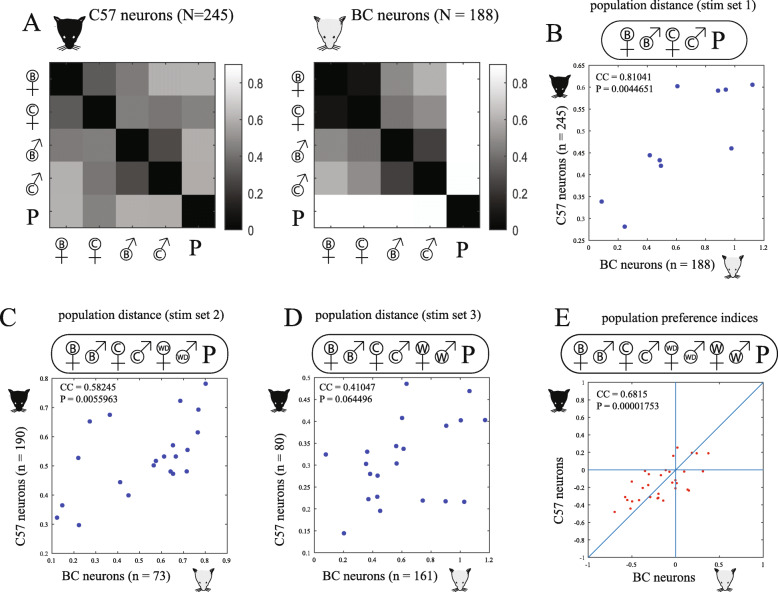
Comparison of responses in each of the two strains. **a** Population-level correlation *distance* matrices (i.e., low correlations are associated with large distances) for each of the 5 stimuli in set 1, for neurons recorded in C57 and BC mice. **b** Correlation between pairwise correlation distances, that is, between corresponding squares in the matrices in **a**. **c** Like **b** but for stimulus set 2 which includes more stimuli (see Fig. 1c). **d** Like **b** but for stimulus set 3. **e** Correlation between population pairwise preference indices for all stimulus pairs except for the four comparisons between wild and wild-derived stimuli, which were never presented in a single stimulus set (resulting in a total of 32 pairwise preference indices: (9•8/2) – 4 = 32, see Fig. 1c). Sample sizes vary for different stimulus pairs but are at least as large as those in panels **b**–**d**. See Additional File 3 for the same analysis using alternative population distance metrics

To assess the relationship between response patterns to the different stimuli, we examined population-level responses. For this analysis, we used the actual response data without normalization (see Additional File 1). As a distance measure, we applied the correlation distance, defined as one minus the correlation coefficient [28, 30]. We note that the correlation distance metric, as well as other metrics that we employ in our analyses (Additional File 3), do not simply sum the activity of individual neurons, but rather take into account the array of response profiles of all individual neurons to each of the stimuli. Distance matrices calculated from neurons from each of the two strains are shown in Fig. 4a. The matrices demonstrate that, as expected [28], distances among same-sex stimuli are smallest, while distances between the various mouse stimuli are smaller than between mouse and predator stimuli. To directly address the question that motivated this analysis, we examined the correlations between the pairwise population response distances across the two recipient strains. With 5 stimuli this yields a total of 10 unique pairwise comparisons. As shown in Fig. 4b, the linear correlation between the two recipient strains’ distances is high (0.81) and very unlikely to arise by chance (*p* < 0.005). This analysis indicates that despite any source of strain-related differences in stimulus detection and processing, there exists a striking match between their population-level representations at the level of the AOB.

To further validate these findings, we extended the analysis with two additional stimulus sets. In one set (set 2, Fig. 1c), we added male and female stimuli from wild-derived mice. These mice, from the *Mus musculus domesticus* subspecies, were shown in a previous behavioral study to differ significantly from inbred mice [25]. Specifically, wild-derived mice displayed increased anxiety and corticosterone levels, and in contrast to lab mice, presented inter-female aggression and pup-directed aggression in sexually-naïve females. Given 7 stimuli, there are 21 unique pairwise comparisons. As shown in Fig. 4c, the distances among stimuli are still positively and significantly correlated among the two strains (*P* < 0.001). In the other stimulus set (set 3 in Fig. 1c), we used urine collected from wild mice, from the *Mus musculus musculus* subspecies [26] (see the “Methods” section). Here too, the correlation was positive (Fig. 4d), although the agreement among the two strains was not as high and the correlation was not significant (*p* = ~0.06). The lower correlations observed in the expanded datasets could reflect diverging representations of these “wilder” stimuli across the two strains (see the “Discussion” section), but may also be affected by the fact that the latter comparisons (Fig. 4c, d) involve considerably smaller datasets (73 BC & 190 C57 neurons for stimulus set 2, 161 BC & 80 C57 neurons for set 3, compared to 188 BC & 245 C57 neurons for set 1 (Fig. 4b, see Additional File 4 for a statistical analysis of the effect of sample size on the observed correlations).

### High correlations across strains in relative response magnitude

Any comparison of response similarity is based on metrics derived from neuronal activity. Despite recent progress, the identity of the relevant metrics in the olfactory bulb (particularly in the AOB) remains largely unknown [31–37]. Importantly, our conclusions remain valid also with the application of other population distance metrics (Additional File 3). In addition to those metrics, another intuitive metric is the summed activity, which was shown to be highly informative about stimulus identity in the main olfactory bulb [38]. Thus, we next tested if the two strains yield similar representations of stimuli using this metric. Our entire dataset includes 9 different stimuli, which yield a total of 32 stimulus pairs (although no single neuron was tested with all stimuli, we were able to compare each pair of stimuli using the neurons that were exposed to both stimuli in that pair). For each neuron, and for each pair of stimuli, we calculated a *preference index* (see the “Methods” section). The index ranges between −1 and 1, with 0 denoting equal responses to the two stimuli. Then, we compared the mean values of these preference indices between the two strains. As shown in Fig. 4e, the correlation between the recipient strains is positive (0.68) and significant (*p* < 0.00002).

Taken together, the results from the foregoing analyses addressed the first interpretation of the question, “Do all mice smell the same?”, namely, whether different mice maintain similar representations of chemosensory space. With the obvious limitation of using only male mice from two distinct strains, the answer to this question is positive. More precisely, we have demonstrated a high degree of correspondence in the manner by which behaviorally relevant stimuli are mapped at the AOB level by two strains that are genetically separated, and likely expressing significantly different vomeronasal sensory neuron arrays (see the “Discussion” section).

### AOB representations of inbred and wild mouse stimuli

**Fig. 5.**
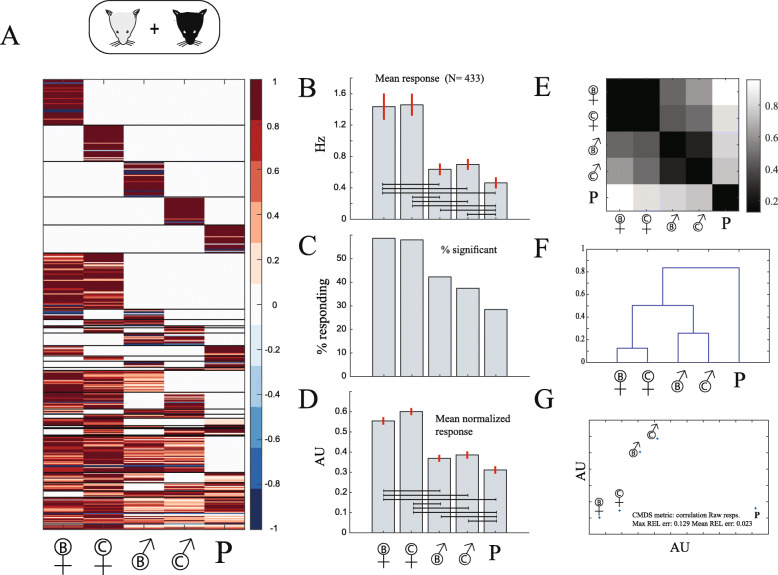
Basic stimulus responses for an integrated dataset. **a** Normalized response matrix (combining the matrices shown in Fig. 3a). **b** Mean response per stimulus across all neurons in the combined dataset. **c** Percent of significant responses per stimulus. **d** Normalized responses to each of the stimuli. **e** Correlation distances between stimulus pairs in the combined dataset. **f** Hierarchical clustering tree of population responses to each of the stimuli, based on the correlation distance. **g** Two-dimensional representation of distances using classical multidimensional scaling of the response distance. In panels **b** and **d**, horizontal lines represent significant differences among stimulus pairs using a one-way non-parametric ANOVA (Kruskal-Wallis test). Broken lines indicate *p* values below 0.05, while solid lines indicate values below 0.01. See Additional File 2 for specific *p* values for this and all other pairwise comparisons. Units in **g** are arbitrary, but the scaling is equal for both axes

We now address the second interpretation of the manuscript’s title. Specifically, we compare responses elicited by stimuli from inbred vs. wild mice. In other words, we ask whether they elicit similar AOB responses. Based on our previous analyses, and specifically the similarity in AOB responses between the two strains, for all subsequent analyses, we combine responses from the two subject strains. To set the baseline, we first test responses to inbred stimuli (stimulus set 1). Our pooled dataset includes recordings from 433 units (245 C57 neurons and 188 BC neurons). The combined (normalized and significant) response matrix is shown in Fig. 5a. Figure 5b shows the mean response magnitude over the entire population of recorded neurons to these five stimuli. We note again the prominently stronger response magnitude to female stimuli, which is reflected also in the fraction of responding units (Fig. 5c), and the mean normalized responses (Fig. 5d). Population-level distances (using the correlation distance) are shown in Fig. 5e. Consistent with previous analyses, similarity in activity patterns reflect ethologically relevant categories, as seen using hierarchical clustering (Fig. 5f) as well as multidimensional scaling (Fig. 5g) [28, 30].

**Fig. 6.**
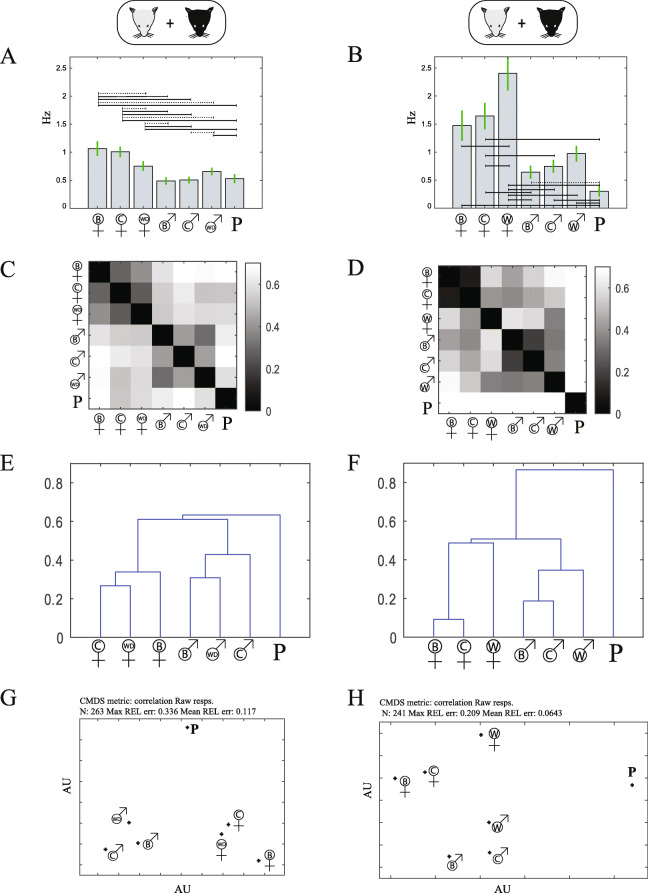
Population responses to wild-derived and wild mouse urine stimuli. **a**, **b** Mean responses across the population for stimuli including wild-derived (A: stimulus set 2, *n* = 263) and wild (B: stimulus set 3, *n* = 241) mouse stimuli. Horizontal lines represent significant differences among stimulus pairs using a one-way non-parametric ANOVA (Kruskal-Wallis test). Broken lines indicate *p* values below 0.05, while solid lines indicate *p* values below 0.01. See Additional File 2 for specific *p* values for this and all other pairwise comparisons. **c**, **d** Pairwise correlation distance matrices of population responses for stimulus set 2 (**c**), and 3 (**d**). **e**, **f** Hierarchical clustering trees using correlation distances for stimulus set 2 (**e**) and 3 (**f**). **g**, **h** Distance representation using multidimensional scaling for stimulus set 2 (**g**) and set 3 (**h**). Units are arbitrary, but in each plot, axes are scaled equally

Next, we analyze responses to the urine of wild-derived mice (263 neurons, BC: 73 neurons, 11 sessions from 8 mice, C57: 190 neurons, 14 sessions from 10 mice). Figure 6a shows that (despite our initial expectation) AOB responses to wild-derived stimuli are not stronger than to inbred stimuli. Likewise, measures of population activity (Fig. 6c, e, and g) indicate that responses to wild-derived urine are similar to those from inbred stimuli: responses cluster according to stimulus sex (regardless of lineage), with predator urine eliciting distinct responses from all murine stimuli. In fact, considering population-level activity patterns, for a given sex, the distance between the two inbred strains is larger than their distances from the wild-derived stimulus (Fig. 6e, g).

Finally, we analyze responses to urine collected from direct offspring of wild-caught mice (*n* = 241 neurons, C57: 80 neurons, 6 sessions in 4 mice, BC: 161 neurons, 9 sessions from 6 mice). These mice are the first lab-bred generation of wild-caught *Mus musculus musculus* mice. They were caught in house shelters and agricultural buildings near Prague and transferred to the animal facility where they gave birth to our stimulus donors [26]. Unlike responses to wild-derived mice, here a clear difference emerges between wild and inbred derived stimuli. First, for both sexes, the response strength elicited by wild stimuli is higher than that evoked by inbred stimuli (Fig. 6b). Notably, the differences are larger (and only significant) for females rather than for male urine. This is somewhat surprising since differences in levels of major urinary proteins, a class of VNS ligands that are known to be higher in wild mice as compared to inbred mice [18, 39], are expected to be more prominent in males. Yet, despite the differences, response intensities are within the same order of magnitude, and while wild male urine does elicit stronger responses than inbred male urine, these do not surpass responses to inbred female stimuli. Second, population-level analyses (Fig. 6d, f, h), which take into account not only how many neurons, but also how activity is distributed across the neuronal population, show a larger distinction between wild and inbred stimuli. While all male and all female stimuli still group together, wild urine, especially from female mice, appears to be distinctly represented by inbred stimuli of the same sex. This is notable, since these wild mice are from a *Mus musculus musculus* species, whereas the wild-derived mice are from the *Mus musculus domesticus* subspecies and thus closer in lineage to the two inbred strains used here [40].

### Sexually dimorphic stimulus strength in inbred and wild strains

Following the general comparison of wild and inbred stimuli, we next study another potential effect of laboratory inbreeding. Specifically, we hypothesized that under laboratory conditions, the effects of sexual selection will be diminished, leading (among other things) to diminished dimorphism in urinary secretions. Furthermore, we speculated that explicit or implicit selection in confined enclosures may favor the attenuation of some masculine or feminine features (see the “Discussion” section).

**Fig. 7.**
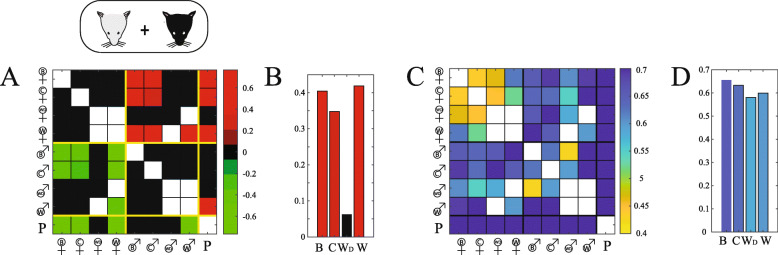
Analysis of sexual dimorphism in secretions from different strains. **a** Preference indices for all combinations of stimulus pairs. Black squares denote indices that are not significantly different from zero. White squares represent comparisons that cannot be made using our dataset since no neurons were tested with both stimuli. **b** The four pairwise indices comparing male and female urine from each of the four strains (BALB/c, C57Bl/6, wild-derived, and wild). Data and colors are the same as for the corresponding squares in **a**. **c** Fraction of selective neurons for each pair of stimuli. A selective neuron shows a significant response to either of the two stimuli in a pair, but not to both. A fraction of 1 corresponds to a scenario where all neurons respond to one of the stimuli but not to both, while a fraction of 0 corresponds to a scenario where all neurons respond to both stimuli. **d** Highlight of the 4 values in **c** that represent male to female comparisons within each of the 4 tested strains

To that end, we calculated the ratio of response strengths for all stimulus pairs across the neuronal population, using the summed population signal (similar, but not identical to the *preference indices* used in Fig. 4e, see the “Methods” section). This analysis is shown in Fig. 7a, where each square corresponds to one pairwise comparison. Note that non-significant differences (see the “Methods” section) are indicated by black squares, while stimulus pairs that were not presented to the same set of neurons cannot be compared and are shown in white. Thus, only the colored squares indicate meaningful differences. The matrix shows that the population response magnitude to all male stimuli is overall similar, as is the case for all female stimuli, and those female stimuli generally elicit stronger responses than male stimuli. To highlight the extent of sexual dimorphism within secretions of the different strains, we show in Fig. 7b only male-to-female comparisons within a strain**,** where positive values represent stronger responses to female cues. For each of the four strains, female urine elicits stronger responses than male stimuli. Somewhat surprisingly, for wild-derived (but not wild) mice, the difference is very small and not significantly different from zero. This is consistent with the observation that wild-derived female urine elicits weaker responses, whereas wild-derived male urine elicits stronger responses than their inbred counterparts (Fig. 6a).

Another indication of differences in the representation of stimulus pairs is given by the fraction of neurons that respond selectively to each of the individual stimuli (rather than to both). This can be interpreted as a measure of the discriminability by the neuronal population to each pair of stimuli. As shown in Fig. 7c, female-to-female comparisons stand out as associated with less selective neurons, as compared to male-male comparisons and female-male comparisons. Here too, to highlight the degree of sexual dimorphism, we plotted the subset of male-to-female comparisons within a strain (Fig. 7d). This representation shows that, for all strains, the fraction of sex-selective neurons is high: more than half of the responsive neurons are exclusively selective to either male or to female urine. Importantly, however, this fraction is not higher for wild-derived or for wild mice, than it is in inbred mice. Taken together, these analyses indicate that at least for the four strains tested here, there is no reduction in sexual dimorphism of urinary stimuli in inbred mice as compared to wild mice (as expressed by neuronal activity patterns in the AOB). Finally, we note that Fig. 7c reveals that the largest fractions of selective neurons (for pairs of mouse-derived stimuli) involve pairs comprising wild and inbred mouse stimuli. This again highlights the distinct nature of AOB response patterns elicited by wild mouse stimuli.

## Discussion

In this study, we addressed two facets of the question *do all mice smell the same*? First, we showed that despite various potential sources of variability, stimulus representations across two strains are very similar. Then, we showed that wild-derived and wild mouse stimuli elicit responses that are qualitatively similar to those elicited by inbred mouse strains. Thus, the short answer to both interpretations of this question is *yes*. However, there are subtleties and potential caveats to this conclusion. Below, we discuss the significance of our findings as well as several limitations of this study.

### Comparison between secretions of wild and inbred mice

One goal of this study was to explore differences between secretions of wild and inbred mice. This is an important question as numerous studies of chemosensory processing in mice were made using inbred stimulus donors (see [3] and references therein). Yet, it is clearly feasible that changes in both the sensory apparatus and in chemical secretions could enhance reproductive success in the laboratory environment. For example, male mice which emit large amounts of cues that elicit aggression towards them might be removed from breeding cages due to injury. Or, to give another example, the presence of pregnancy blocking signals [41, 42] may favor reduced chemosensory sensitivity in females. These examples are speculative and merely serve to illustrate that intentional and unintentional selection could shape chemical advertising and signal processing in inbred mice. Indeed, studies of major urinary proteins have shown considerable differences between inbred and wild mice [18]. As such, our study provides an important and overall reassuring conclusion, namely that responses to wild and inbred stimuli are similar, in terms of response intensity (magnitude and number of responding cells), population-level representations, and sexual dimorphism. Responses to male and female wild secretions cluster with those of male and female inbred stimuli, implying that despite many generations of inbreeding, domestication in a laboratory environment, and the significant disparity between the two wild strains used here, AOB responses maintain the ability to capture the key ethological features in these stimuli. Furthermore, our analysis shows that domestication in the laboratory did not lead to an obvious loss of dimorphism in urinary composition.

Importantly, beyond these first-order similarities, there are interesting differences between wild-derived, wild, and inbred stimuli (Fig. 6). Indeed, we observe a substantial fraction of neurons that are selective to wild rather than inbred stimuli (Fig. 7). Response patterns to wild stimuli from the *Mus musculus musculus* subspecies are clearly distinct from responses to inbred strains, whereas the differences between inbred stimuli and wild-derived *Mus musculus domesticus* mice appear smaller. This is in good agreement with the larger evolutionary distance between the *musculus musculus* subspecies and inbred mice. The two subspecies diverged approximately half a million years ago [43], and offspring between *domesticus* and *musculus* subspecies are sterile. Thus, breeding avoidance based on chemosensory detection could provide an adaptive mechanism to avoid the costs of infertile mating. At least with respect to major urinary proteins, the two sub-species do indeed show distinct expression profiles [44–46], and these can be employed for subspecies detection. An important remaining goal for future studies is to identify which urinary constituents account for the differential responses observed here.

We note that all stimulus samples were pooled across individuals. Pooling was employed to reduce variability across samples that result from factors such as individual differences and fluctuating physiological states. Because we did not monitor the estrus stage of the female urine donors, female urine mixes likely contain samples across the entire estrus cycle. This is significant as vomeronasal sensory responses to urine may change as a function of the donor’s estrus cycle [34, 47]. While pooling reduces variability, it may also mask interesting aspects of the stimuli, which could distinguish inbred from wild strains. For example, the effects of the estrus cycle and/or individuality on urine composition may be more pronounced in wild as compared to inbred strains.

It should also be noted that in some of our analyses (i.e., for stimulus set 1), we combined responses to urinary samples from different inbred sub-strains. Although the features of responses to the two sub-strains are generally similar (see Additional File 5), combining the responses might have added another source of variability to our stimulus set, due not only to potential genetic differences but also to dietary differences associated with the two providers and animal facilities. Yet, any such differences, if exist, are likely to have weakened rather than to account for our main conclusion. Another inherent factor that could account for differences in urine secretions among each of the groups, and particularly for comparisons between inbred and wild stimuli, is the microbiome [48]. Yet, we stress that in our direct comparison of inbred and wild mice (stimulus set 3), all urine donors were fed with the same diet.

Finally, all the comparisons made here are based on two inbred strains of male mice. It is possible that receptors expressed in wild mice would reveal further differences between inbred and wild secretions. This would be the case if inbreeding led to the loss of vomeronasal sensory receptors that are specifically tuned to wild mouse urinary components. Similarly, it remains to be seen if the stereotypy observed between the two tested inbred strains will generalize to other inbred, outbred, or wild mouse strains. These latter considerations underscore the importance of recording from wild mice recipients. While this endeavor presents significant practical challenges, it remains an important goal for future studies.

### Stimulus mapping and stereotypy in the AOB

The VNS is best known for its role in mediating innate behaviors that include mating, parenting, fighting, and avoiding dangers [14, 30, 49–59]. The notion of innate behaviors is commonly associated with a hardwired ordered organization, which would allow a predetermined mapping between stimuli and responses. Yet, our current understanding of the organization of the VNS, and specifically the AOB, suggests that, at least in comparison to the main olfactory bulb, its organization is more variable between individuals. Specifically, this is based on the glomerular innervation of sensory neurons and MTCs [9, 60, 61] and the functional mapping of glomerular responses [62]. Further complicating the picture are the systematic differences among strains at the sensory neuron level [5, 7, 8]. It is these considerations that led us to test similarity in AOB representations between different mouse strains, treated here as proxies for distinct individuals.

An important question is the degree to which these strains actually differ in vomeronasal stimulus processing. In other words, how large are the variations among individuals within each strain and between the two strains. This can be influenced by both peripheral and central aspects, the former of which are considerably easier to analyze. The most comprehensive analysis of vomeronasal receptor (VR) repertoires across multiple strains of inbred, wild-derived, and wild mice has revealed significant differences in sequence compared to the C57BL/6 reference genome [17]. This analysis has shown 301 single-nucleotide polymorphisms (SNPs) among the 202 VRs that could be compared between the BALB/cJ strain and the C57BL/6 genome; many (61%) of which were non-synonymous. The total number of SNPs was much larger in wild-derived mice from the *musculus* subspecies (PWK/PhJ), but not in wild-derived mice from the *domesticus* subspecies (WSB/EiJ), the same sub-species of both inbred strains used here. Notably, the fraction of private SNPs (unique to a given strain) was considerably higher in the wild-derived mice (~18% and 36% for *domesticus* and *musculus* derived strains, respectively, as compared to <1% for BALB/cJ mice). Likewise, the combined number of duplicated, deleted, truncated, and frame-shifted genes was higher in *musculus*-derived wild mice (~12%) in comparison to BALB/c or the *domesticus* wild-derived mice (~4% in both for both strains).

Notably, in addition to these differences at the genomic level, it was shown that strain-to-strain variation in VR expression patterns is prominent and very likely to influence vomeronasal processing [8]. Taken together, these and other analyses of genetic differences in vomeronasal receptor genes across mouse strains [63–68] suggest that vomeronasal stimulus detection in mice is strongly strain-dependent.

Our experimental approach cannot reveal whether there is a stereotypical representation of stimuli across the spatial extent of the AOB, as has been demonstrated in the main olfactory bulb [69–71]. That is, whether any particular stimulus, in itself, elicits similar response patterns in the AOBs of different individuals. However, using both a simple measure of mean response strength, and more nuanced population-level comparisons, we showed that AOB representations are positively and significantly correlated between the two strains. Namely, pairs of stimuli that elicit similar responses in mice from one strain tend to elicit similar responses in the other strain.

One obvious limitation in our study is the use of only two subject groups, namely male mice from two distinct inbred strains. We used males for simplicity, and because previous studies suggest that within a strain, there is very little sexual dimorphism in olfactory/vomeronasal receptor expression patterns [5, 6, 8] and AOB responses [28, 30]. However, recordings in female mice are required to test the generalizability of these assumptions.

Regardless of the extent of randomness vs. predefined order in the AOB, our study reveals a large degree of stereotypy in the manner by which ethologically meaningful vomeronasal stimuli are represented. It is noteworthy that such stereotypy is observed not only despite the biological variability across strains and individuals and their life history, but also the experimental variability associated with a random sampling of AOB units across individuals, and across different divisions of the AOB (which cannot be easily distinguished in our recordings). In other words, even a rather sparse random sampling of AOB MTCs as conducted here can reveal similar patterns of stimulus mapping across strains. It is tempting to speculate that the manner by which AOB MTCs sample glomeruli (whether randomly or by conforming to some rules) can account for some of the similarity across the strains that we observe here. Further analysis and modeling of VSN response patterns and their connectivity with AOB MTCs could shed light on the neuronal underpinnings of our observations.

Taking a broader view, our study touched upon a fundamental problem in biology, namely how stereotypy is achieved despite randomness [72]. Notably, at least in human olfaction, beyond relatively subtle individual differences, there are clear similarities in the perception of chemosensory space across individuals [73, 74]. When the meaning of sensory stimuli is predetermined, labeled lines can be used to map them to behavior, and this is indeed the case in some invertebrates [75–79] and vertebrates [14]. However, when more complex nervous systems need to mediate responses to stimuli that are themselves more complex and variable, it is less clear how stereotypical representations can be achieved. Here, we studied one important instance of this broad topic in the context of chemosensation, namely whether different individuals maintain consistent mappings of their external world. In many ways, the problem studied here in the context of the VNS is related to the creation of consistent representations in main olfactory system structures (such as the piriform cortex) across individuals [80], or across the left and right hemispheres of an individual [81].

### Finer discriminations and the potential role of learning in the AOB

The stimulus sets that we used do not cover the entire coding capacity of the VNS, and the consistent mapping observed here across strains may be achieved with a far simpler structure than the AOB. More specifically, the stereotypy observed here applies to stimuli spanning broad ethological categories. Just like humans generally agree on broad perceptual categories and differ with respect to finer distinctions [73, 74, 82], the similarity that we observed may break down when stimulus sets representing subtler distinctions are used (e.g., urine samples from two individuals from the same strain). What then are these subtler distinctions, which the AOB (with its apparently random organization) must allow? We suggest that the AOB plays a key role in representing individuals—whose secretions are often best described as *signature mixtures* rather than pheromones [19, 20, 83, 84]—and their specific physiological states [34, 85]. Because the ethological relevance of each individual must be learned through experience and may change with time, behavioral responses to stimuli must be plastic. Supporting this general notion, recent studies have highlighted the role of the VNS in mediating flexible responses [3, 86–88]. The specific structure of the AOB may be suitable for associating among different components, as has been shown for the main olfactory cortex [89], yet at an earlier stage of processing.

## Conclusions

We found that neuronal representations of ethologically relevant urine stimuli at the level of the AOB are similar for (male) mice from two different inbred strains, despite any potential differences in receptor repertoires or apparently random wiring at the level of the AOB. In addition, we found that wild-derived mouse stimuli elicit responses that, although not identical, are nevertheless qualitatively similar to those from commonly used inbred strains. This validates the use of stimuli from inbred mouse donors, yet also calls for a more detailed comparative analysis of the chemical features of inbred and wild mouse stimuli, and the responses elicited by them.

## Methods

### Mice

Recordings were made from adult (8–12 weeks old) BALB/c OLAHSD (BC), and C57BL/6 JRCCHSD (C57) male mice purchased from the Harlan Laboratories (Jerusalem). All experimental procedures were approved by the ethical committee of the Hebrew University Medical School. Urine collection was also performed at the Weizmann Institute (Rehovot, IL) and Charles University (Prague, Czech Republic) according to each institute’s ethical committee’s guidelines.

### Stimuli

For inbred stimuli used in stimulus set 2 (see Fig. 1c), we used mice that were purchased from the Harlan Laboratories (Jerusalem). Fresh urine samples were collected from adult male and female mice of the BALB/c OLAHSD and C57BL/6JRCCHSD strains. Stimuli were collected by placing mice on a plastic bag and gently pressing the abdomen to encourage urination. Urine was immediately collected to a microcentrifuge tube and kept in liquid nitrogen until freezing at −80°C. For wild-derived stimuli (stimulus set 2), we used wild-derived mice, originating from the fields of Idaho, USA, and bred for several generations in the animal facility of the Weizmann Institute (Rehovot, Israel), with strict prevention of inbreeding [25, 90]. Here too, stimuli were collected by placing mice on a plastic bag and gently pressing the abdomen to encourage urination. Urine was immediately collected to a microcentrifuge tube and kept in liquid nitrogen until freezing at −80°C. Inbred mouse urine for stimulus set 3 was collected from inbred mice (C57BL/6NCrl and BALB/cAnNCr) purchased from pathogen-free facility of the Institute of Molecular Genetics (Czech Academy of Sciences in Prague). Wild mice stimuli in stimulus set 3 were from mice caught in house shelters and agricultural buildings near Prague (Czechia) that were transferred to the animal facility. Their offspring, which are the urine donors for this stimulus set, were fed on the same diet as the two inbred strains in this stimulus set. Food and water for all these strains were provided ad libitum and under stable conditions (i.e., 13:11 h, D:N, temperature t=23°C). The urine was collected by holding a mouse above a sterile glass sheet. Predator urine (for all stimulus sets) included bobcat and fox urine from Predatorpee (https://www.predatorpeestore.com) as well as rat urine from rats purchased in the Harlan Laboratories (Jerusalem). All three predator urine sources were combined in equal volumes and diluted 1:10 in Ringer’s. For each mouse stimulus, to minimize individual to individual variability, we created pools from 8 individuals of each strain. Then, the urine was diluted 1:10 in Ringer's solution, divided into aliquots and stored in −80°C.

Note that inbred stimuli in stimulus set 1 (with stimuli from both the Jerusalem and the Prague labs) comprised stimuli from inbred mice from different sub-strains (see Additional File 4). The estrus stage was not determined in wild nor in inbred mice. However, pooling across mouse samples (across 8 individuals in each stimulus set) is designed to reduce variability due to this important factor.

### Surgical preparation

All recordings were made in the AOB of anesthetized mice, using procedures and an experimental preparation described in detail in [15]. Briefly, anesthesia was introduced with the intraperitoneal injection of a ketamine-xylazine mix (xylazine = 10mg/kg and ketamine = 100 mg/kg) or alternatively, 3% isoflurane mixed with oxygen gas in an anesthetic chamber. After placing the mouse on a stereotaxic stage, anesthesia was maintained with ~1% isoflurane and monitored according to the heart rate and by testing the foot withdrawal reflex. A tracheotomy was performed with a polyethylene tube and a cuff electrode was placed around the sympathetic nerve trunk. Incisions were closed with veterinary glue after which the mouse was placed in custom-built stereotaxic apparatus. A craniotomy was made immediately rostral to the rhinal sinus. The dura was removed around the penetration site and electrodes were advanced into the AOB at an angle of ~30° with a manual micromanipulator (Sutter Instruments, Novato, CA). All recordings were made with acute 32-channel probes purchased from Neuronexus (Ann Arbor, MI). The following configurations were used: 4 shanks x 8 sites per shank, 5-mm shank depth, 50- or 100-μm inter-site distance, 200-μm inter-shank distance, and 177-μm^2^ or 413-μm^2^ site area. Before penetration, electrodes were dipped in fluorescent dye (DiI, Invitrogen) to allow subsequent confirmation of electrode placement within the AOB external cell layer, which contains the principal cells of the AOB [3].

### Stimulus delivery

During each trial, 2 μl of all the stimuli was applied directly into the left nostril (which was also the side of cuff electrode placement). After a delay of 20 s, a square wave stimulation train (duration 1.6 s; current 120 μA; frequency 30 Hz) was delivered through the cuff electrode to the sympathetic nerve trunk (SNT) to induce VNO pumping and stimulus entry to the VNO lumen (SNT stimulation). Following another delay of 40 s, the nasal cavity and VNO were flushed with 1–2 ml of Ringer’s solution, which flowed from the nasal cavity and was drained via the nasopalatine duct (see [15]) using a solenoid-controlled suction tube. The flushing procedure was 50s long and included a single sympathetic trunk stimulation to facilitate stimulus elimination from the VNO lumen. An additional 10 s period separated trials. In each session, several different stimuli were presented in a pseudorandom order (see Fig. 1c), at least four, and typically five times per stimulus. The experiments were controlled using custom-written MATLAB programs (MathWorks).

### Data collection and unit selection

Neuronal data were recorded using an INTAN board (RHD2000 V1, Intan Technologies) integrated with a data acquisition board (USB-6343, National Instruments). Signals were sampled at 25 kHz and bandpass filtered (300–5000 Hz). Spike waveforms were extracted using custom-written MATLAB code. Spikes were sorted automatically using Klusta-Kwik [91] and then manually verified and adjusted using Klusters [92]. Spike clusters were evaluated by their spike shapes, projection on principal component space (calculated for each session individually), and autocorrelation functions. A spike cluster was designated as a single unit if it showed a distinct spike shape, was fully separable from both the origin (noise) and other clusters along with at least one principal component projection, and if the interspike interval histogram demonstrated a clear trough around time 0 of at least 10 ms. Clusters not meeting these criteria were designated as multi-units and were excluded from the analysis. Contaminated single units were clusters that are mostly well separated from other clusters, but were likely to include minor contributions from other units. Both single units and contaminated single units were used in this manuscript.

We have previously described our observation that in some cases, stimulus application is sufficient to induce a neuronal response before sympathetic nerve trunk stimulation [15, 28]. Such stimulus application-locked responses present genuine VNO-mediated AOB responses and are included in the present analysis, because our intention was not to study the precise temporal features but rather the magnitude of the response. Therefore, in this manuscript, we followed the same convention used in [30], which is to include the entire post application and post stimulation period. This means that some of the responses occur mostly during the application epoch (i.e., after stimulus application and before electrical stimulation), some during the post-stimulation epoch, and some during both periods. Figure 2 shows examples of each of these response types, all of which are clearly vomeronasal-mediated responses in the AOB. The tendency for different response types is mostly a feature of the specific experimental preparation and likely the anesthesia level of the subject mouse.

To be included in the analysis, single units had to exhibit significant responses to at least one of the tested stimuli. A stimulation-locked response is considered significant if the distribution of single-trial firing rates (typically, five single-trial values for each stimulus), quantified for 50 s following stimulus application, is significantly different from the distribution of the pre-stimulus firing rate of the same neuron. The pre-stimulus firing rate distribution is evaluated during the 10 s period before stimulus application and pooled across all trials of all stimuli for the neuron in question. The response of a neuron to a given stimulus is considered significant if these distributions differ at the *p* < 0.05 significance level, determined using ANOVA (MATLAB anova1 function).

### Data analyses

All analyses were conducted using custom-written MATLAB code and built-in MATLAB functions when applicable (e.g., mean, std). After spike sorting, all units and their single-trial responses to each of the stimuli were compiled into a single MATLAB table, which was used as a basis for all subsequent analyses. Population response matrices such as shown in Fig. 3a (and in other panels) represent the mean firing rate changes following stimulus application (as described above). For each unit, we normalized the maximum to 1 (or to −1 in those cases where the maximal response was a reduction relative to baseline rates) and adjusted responses to all other stimuli according to the same factor. In addition, in these matrices, all non-significant responses were set to 0. We note that these matrices were used only for visualization, while the actual data analysis (unless indicated otherwise) was applied to the raw un-normalized data, without setting non-significant responses to 0. The raw and normalized matrices are shown in Additional File 1. For comparison of response magnitudes to different stimuli, we used non-parametric one-way ANOVA (Kruskal-Wallis), implemented with the Kruskal-Wallis function in MATLAB.

#### Population distance metrics and comparison

Population distance matrices, such as shown in Fig. 4a (and in other panels), were calculated using the *pdist* function in MATLAB. For matrices shown in the main text, we used the *correlation* distance, which is defined as 1 minus the linear correlation coefficient of the corresponding population response vectors. In Additional File 3, we also show the response distance matrices using the *cosine, Euclidean,* and *standard Euclidean* distance measures. The correlation between distances (Fig. 4b–d) was generated by plotting the pairwise population distances for each stimulus pair, according to the neurons recorded in each of the subject strains. Only off-diagonal terms were considered, and each pairwise correlation was considered only once (although the complete symmetric matrices are shown in the panels). In Fig. 4b, each data point represents one pairwise distance from the matrices in Fig. 4a. In Fig. 4c, d, each data point represents one of the pairwise distances in stimulus sets 2 and 3, respectively (the distance matrices themselves are not shown). Population-level distance plots in Additional File 3 were made similarly, but in addition to the correlation distance, we also applied the *cosine*, *Euclidean*, and *standard Euclidean* distance measures. Hierarchical clustering trees (Figs. 5 and 6) were generated using the MATLAB *linkage* function (using the METHOD *average* and distance METRIC *correlation*) and the *dendrogram* function to generate the hierarchical trees. Two-dimensional distance approximations (Figs. 5 and 6) were generated using the MATLAB *cmdscale* function which applies classical multidimensional scaling, with distance relationships created with population correlation distances derived by the *pdist* function. Multidimensional scaling plots were made using the first two dimensions returned by the function.

#### Preference and modulation indices

The preference index for a stimulus pair (A, B) for a given unit is defined as (Ra-Rb)/(Ra+Rb), where Ra and Rb are the unit responses to the two stimuli. For these analyses, all negative responses were set to 0 to keep preference indices within the range −1 and 1. In Fig. 4e, we plot the mean preference indices across all units, for each stimulus pair, for each of the two strains. As can be seen in the data matrices such as in Fig. 3a or Fig. 5a, the vast majority of responses are positive and thus not affected by this correction. The reason that Fig. 4e contains more data points than Fig. 4b–d is that this analysis does not require neurons to be tested with the entire stimulus set, but rather only with the two stimuli within each pair. Thus, unlike the case in Fig. 4b–d, the neuronal populations used for each data point generally differ, depending on the identity of the neurons that were tested with both stimuli. The modulation index (shown in Fig. 7a, b) for a pair of stimuli is defined as Mod index = (R1–R2)/(R1+R2) where R1 and R2 are the mean population responses for stimulus 1 and stimulus 2. Neurons included in each pairwise calculation had a significant response to at least one of the two stimuli. Significance was calculated using a bootstrap approach. Specifically, in each of 10,000 repeats, the entire set of stimulus responses was randomly shuffled within the entire matrix, and the modulation index was calculated. To be considered significant, the actual modulation value had to be at least as large as 0.9984 of the shuffled values. This approach ensures that the modulation index is larger than expected by chance upon a random distribution of responses among the stimuli. In other words, the difference is unlikely to arise by chance under the null hypothesis that both stimuli elicit identical responses. The value of 0.9984 (1–0.05/32) corresponds to a significance level of 0.05 after correction for 32 pairwise comparisons present in our data set. The matrix in Fig. 7c represents the fraction of selective neurons per stimulus pair. That is, the fraction of neurons that are responsive to stimulus 1, or stimulus 2, but not to both. Here, too, we used a similar bootstrapping approach to determine whether the fraction was significantly larger than expected by chance. Here, rather than shuffling the response magnitudes as above, we shuffled the significance designations (that is, binary values indicating whether the neuronal response to each stimulus was significant at the 0.05 level) and used it to determine if the observed fraction of selective neurons was larger than expected under the null hypothesis (according to which there is no difference between the two stimuli). In all cases, the fraction of selective responses was significant according to the 0.05/32 threshold.

## Supplementary Information

**Additional file 1.** PDF file with raw, normalized and normalized and non-significant truncated response matrices, shown for neurons for each of the two strains.

**Additional file 2.** PDF file with details of statistical comparisons of responses for different categories.

**Additional file 3.** PDF file with similarity in responses across the two strains using various distance metrics.

**Additional file 4.** PDF file showing bootstrap analysis to test the effect of sample size on the observed correlation in representations between the two strains (Fig. 4).

**Additional file 5.** PDF file showing a comparison of basic response features to the inbred stimuli from two different sources.

## Data Availability

The datasets used and analyzed during the current study, as well as the analysis codes, are available from the corresponding author on reasonable request.
