# Supplementary figures and images for: Do all mice smell the same? Chemosensory cues from inbred and wild mouse strains elicit stereotypic sensory representations in the accessory olfactory bulb
Source: BMC Biol. 2021 Jun 28;19:133. doi: 10.1186/s12915-021-01064-7 (PMC8240315; doi:10.1186/s12915-021-01064-7)

## Additional Figure 1

BC Subjects (n = 188)

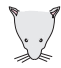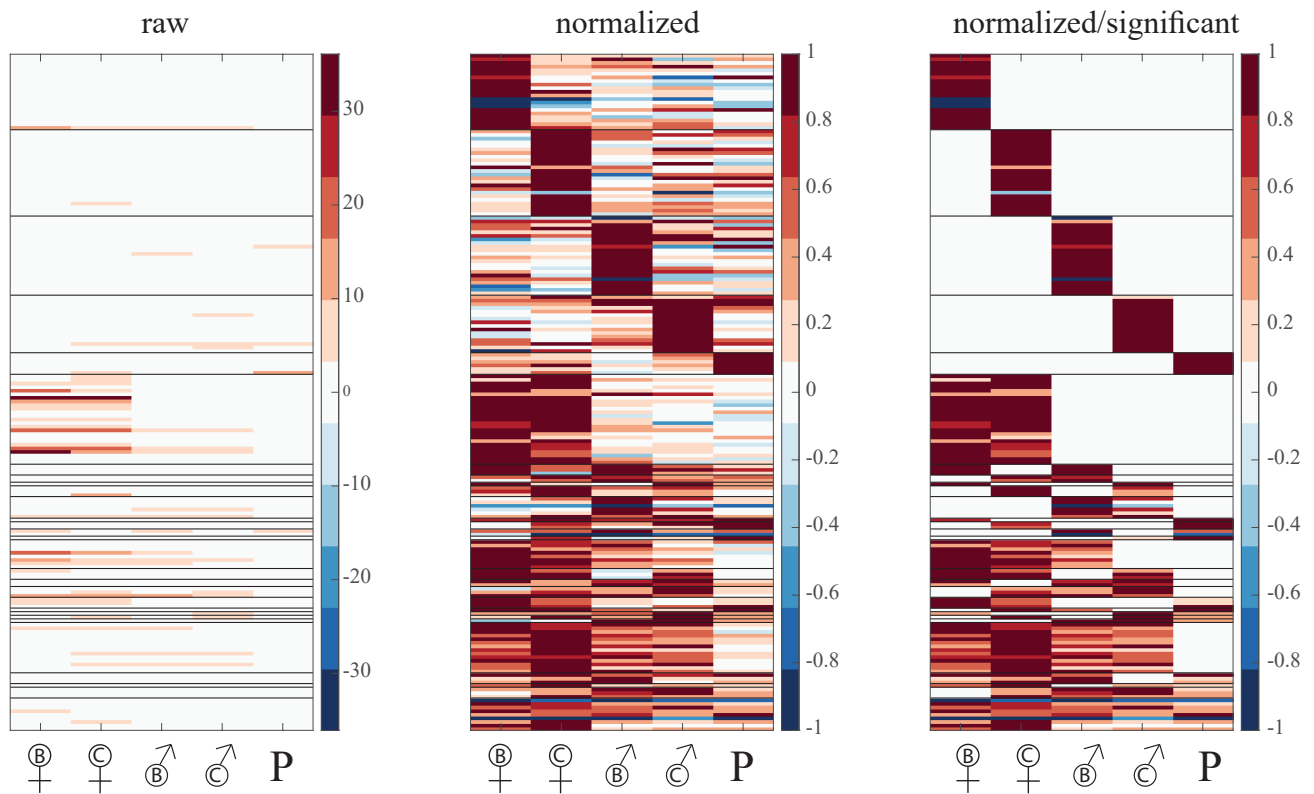

C57 Subjects (n = 245)

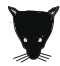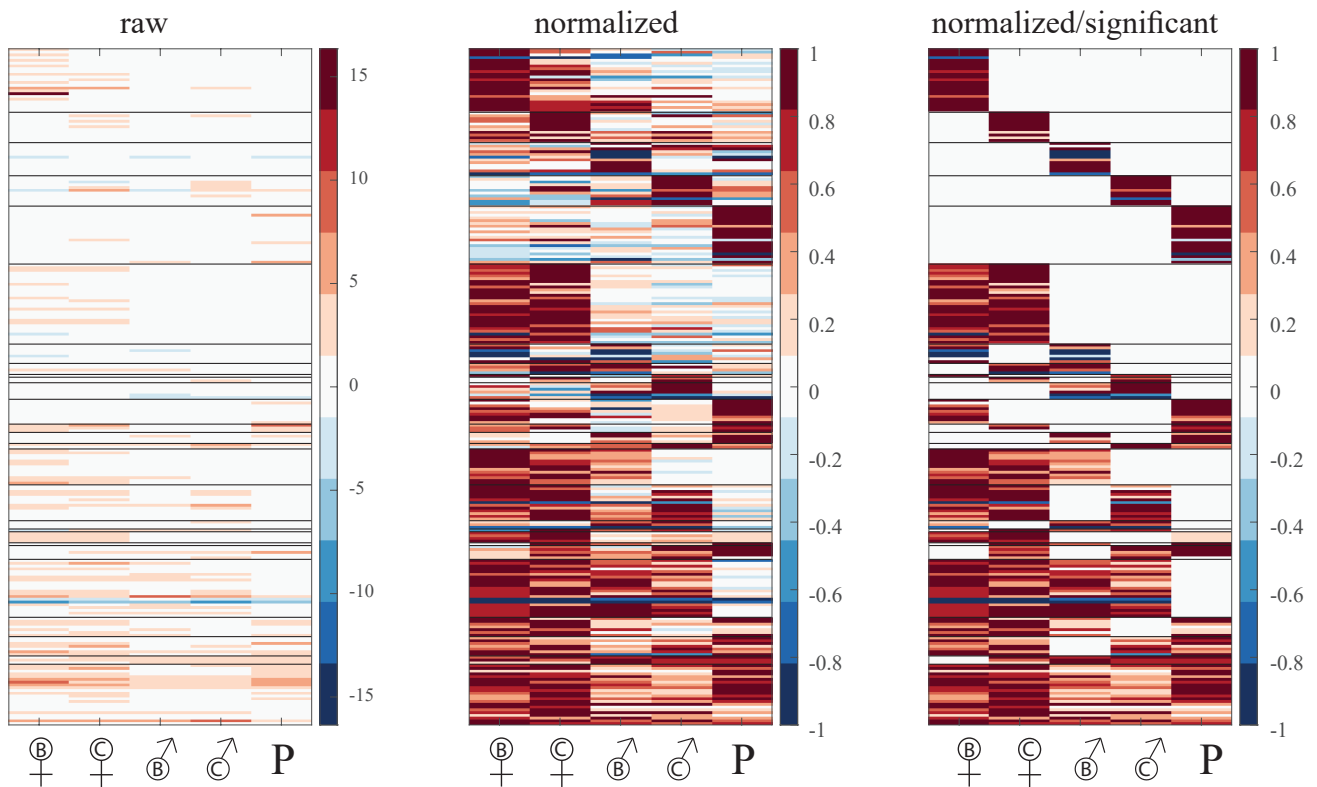

Supplement: Supplementary file 1 — Additional file 1. PDF file with raw, normalized and normalized and non-significant truncated response matrices, shown for neurons for each of the two strains. [file 12915_2021_1064_MOESM1_ESM.pdf]
