# Supplementary material for: Do all mice smell the same? Chemosensory cues from inbred and wild mouse strains elicit stereotypic sensory representations in the accessory olfactory bulb
Source: BMC Biol. 2021 Jun 28;19:133. doi: 10.1186/s12915-021-01064-7 (PMC8240315; doi:10.1186/s12915-021-01064-7)

Additional File 2 – details of statistical comparisons of responses for different categories

Detailed significance testing of all stimulus sets and subject groups shown in the manuscript. All p-values are obtained using a one-way non-parametric ANOVA (Kruskal-Wallis test, kruskalwallis test in MATLAB). For each stimulus set (1,2, or 3 as shown in Figure 1C) and subject group (either BALB/c, C57, or both) we present the results for the raw responses, and the normalized responses. P-values are not corrected for multiple comparisons. P values < 0.05 are highlighted in green. P-values < 0.01 are highlighted in yellow.
The first row in each matrix denotes the p-value for comparison of the 1st stimulus with all the others. The second row denotes the p-value of the 2nd stimulus with all the others. NaN values are included to avoid duplicate values. The results are also shown graphically. The line compares the stimulus in the left side of the line with a stimulus to its right. If there is a red circle, then there is a difference with P<0.01. If there is a black circle, the P<0.05. Some of these values are also shown graphically in the main manuscript.

Stimulus set 1, subject strain: C57

Raw values

9.3592e-01	1.4547e-07	4.1860e-06	5.3071e-08
NaN	1.1221e-07	2.1263e-06	1.9777e-08
NaN	NaN	6.3452e-01	4.7383e-01
NaN	NaN	NaN	2.0839e-01

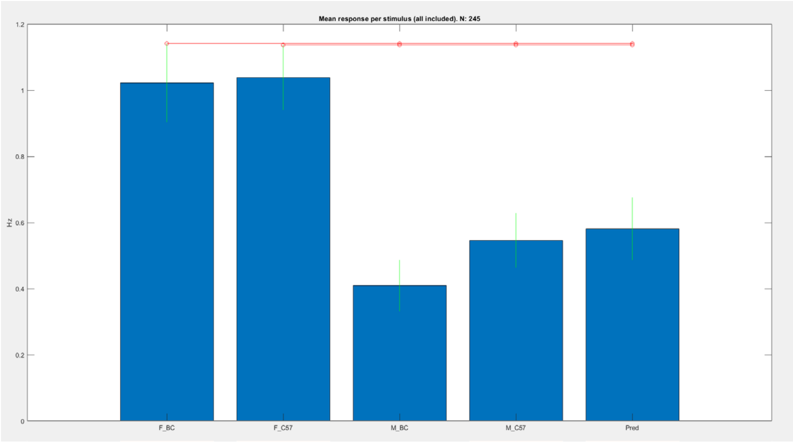

Normalized values

7.3655e-01	2.6599e-14	4.9251e-11	4.6758e-11
NaN	6.3567e-15	9.1342e-12	2.3666e-11
NaN	NaN	4.9809e-01	7.2998e-01
NaN	NaN	NaN	3.1589e-01

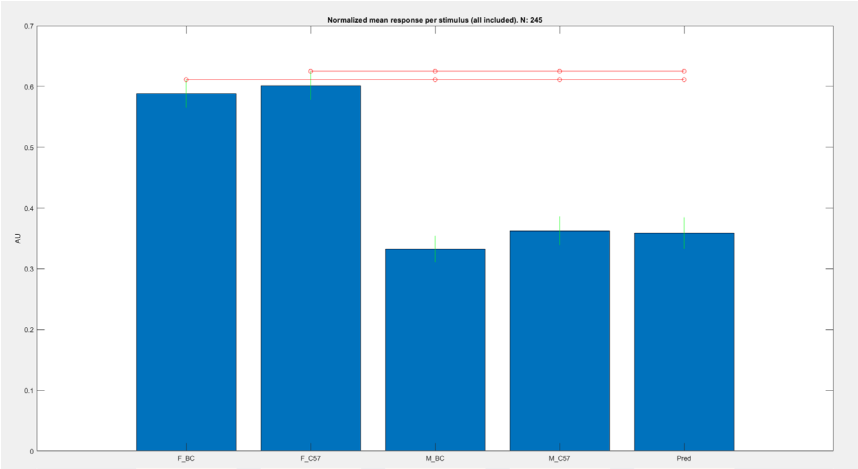

Stimulus set 1; Subject strain: BC.

Raw values

3.4504e-01	2.0621e-01	1.7476e-01	2.4978e-08
NaN	1.9743e-02	1.5933e-02	2.5092e-11
NaN	NaN	9.1837e-01	3.3032e-06
NaN	NaN	NaN	2.7908e-06

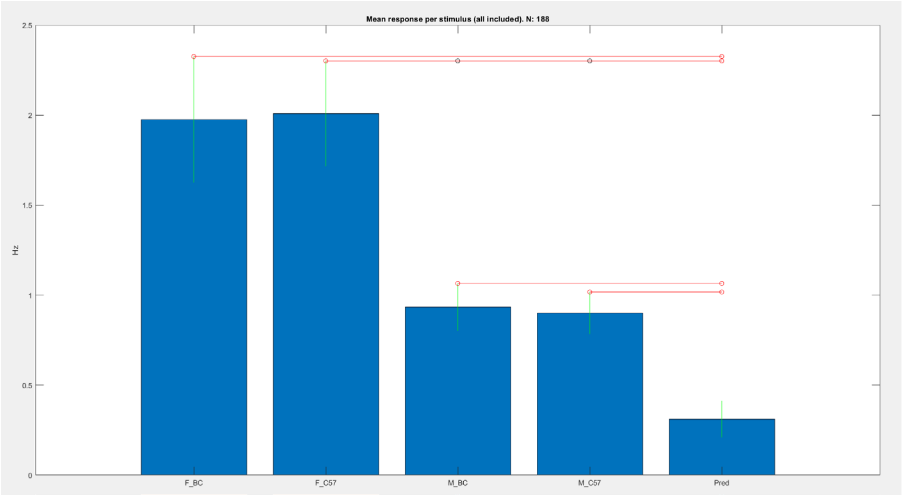

Normalized values

2.0095e-02	6.3847e-02	6.4704e-02	1.1076e-10
NaN	6.2666e-06	4.7858e-06	8.8038e-19
NaN	NaN	9.8934e-01	3.3170e-07
NaN	NaN	NaN	9.9045e-08

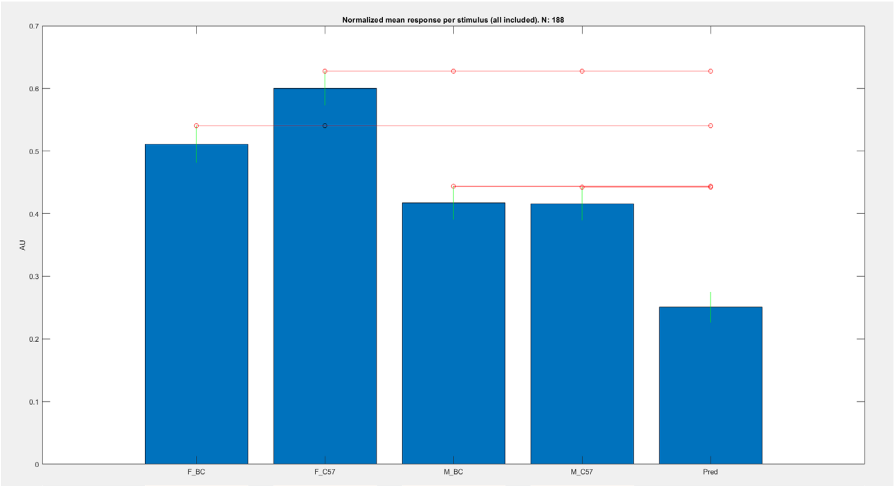

Stimulus set 1, Subject train: C57 + BC:

Raw values

4.8416e-01	2.3820e-06	1.4811e-05	4.2507e-15
NaN	4.4104e-08	2.9735e-07	3.8434e-18
NaN	NaN	6.9812e-01	4.4086e-04
NaN	NaN	NaN	8.3504e-05

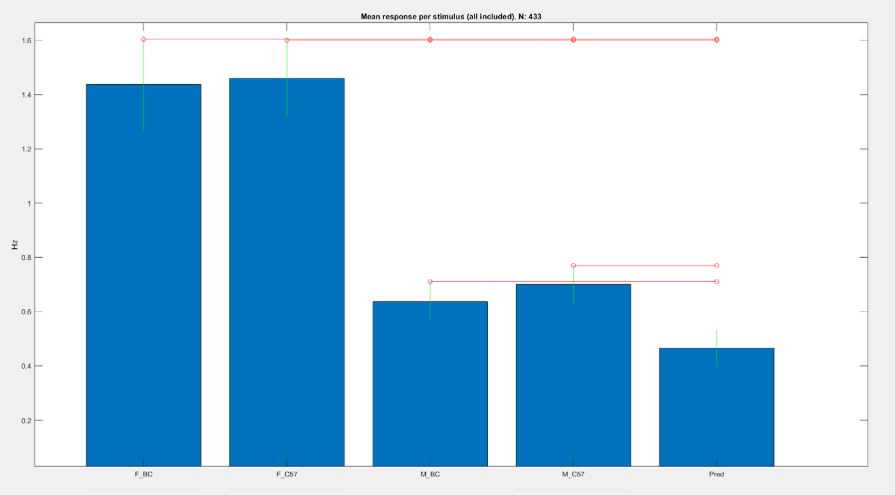

Normalized values

7.5797e-02	3.9937e-12	3.2355e-10	1.9030e-20
NaN	1.0476e-18	3.0783e-16	1.1045e-27
NaN	NaN	5.6825e-01	3.3630e-04
NaN	NaN	NaN	2.8372e-05

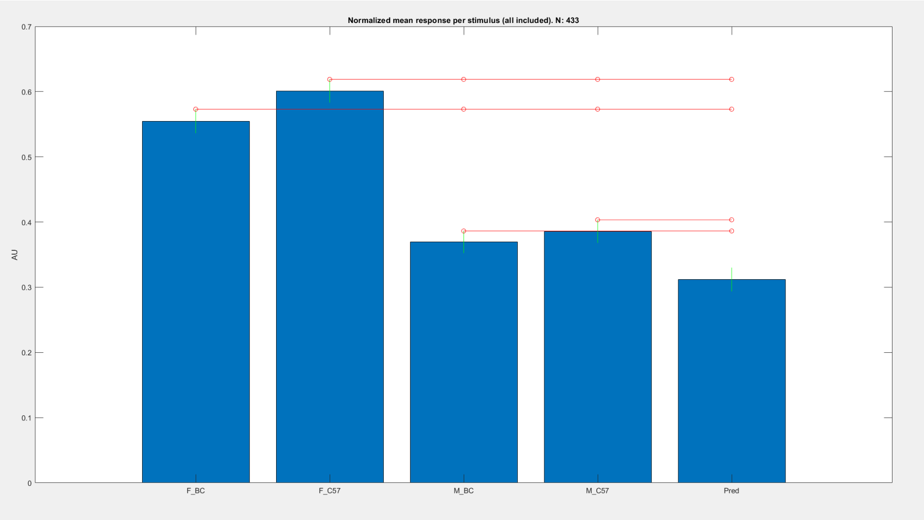

Stimulus set 2. Subject strain: C57 + BC

Raw values

9.6087e-01	3.7801e-02	1.5737e-05	3.1216e-06	1.9384e-02	1.0901e-08
NaN	4.3065e-02	2.0665e-05	3.0740e-06	2.0264e-02	1.3162e-08
NaN	NaN	2.5696e-02	7.0582e-03	7.5800e-01	9.8330e-05
NaN	NaN	NaN	5.7060e-01	6.2060e-02	6.4549e-02
NaN	NaN	NaN	NaN	2.1486e-02	1.9150e-01
NaN	NaN	NaN	NaN	NaN	4.4000e-04

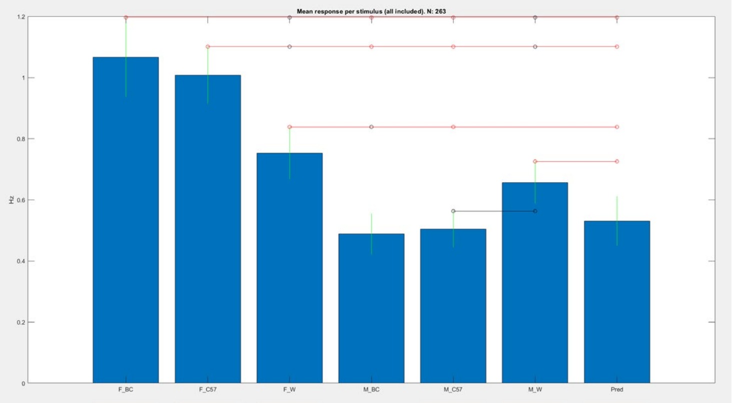

Normalized values

9.5724e-01	1.0245e-04	5.1946e-11	4.6405e-12	1.0716e-05	2.0357e-11
NaN	9.7523e-05	3.4643e-11	2.2034e-12	1.2952e-05	1.1092e-11
NaN	NaN	7.2395e-04	1.0153e-04	3.8758e-01	3.8312e-05
NaN	NaN	NaN	5.2100e-01	1.8045e-02	1.3227e-01
NaN	NaN	NaN	NaN	3.1957e-03	3.3420e-01
NaN	NaN	NaN	NaN	NaN	6.0055e-04

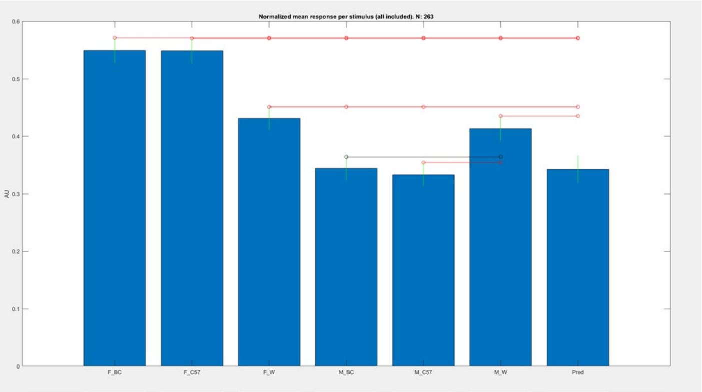

Stimulus set 3. Subject strain: C57 + BC:

Raw responses

9.1777e-02	1.9229e-06	6.0373e-02	2.0299e-01	8.9205e-01	2.1292e-05
NaN	8.0063e-04	1.8636e-04	1.8942e-03	5.8562e-02	1.9942e-10
NaN	NaN	3.8951e-12	1.9480e-10	1.2576e-07	2.2880e-21
NaN	NaN	NaN	4.6047e-01	5.5658e-02	1.0480e-02
NaN	NaN	NaN	NaN	2.3766e-01	1.4616e-03
NaN	NaN	NaN	NaN	NaN	1.1565e-05

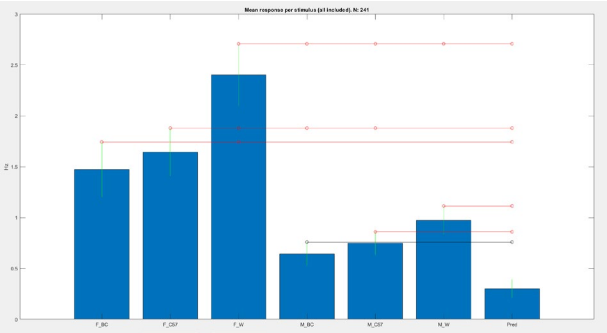

Normalized responses

1.1271e-02	1.4530e-15	1.1145e-02	6.3927e-02	7.6998e-01	5.1659e-08
NaN	3.8553e-12	2.8528e-09	2.0656e-07	5.5768e-04	1.1148e-18
NaN	NaN	1.3474e-26	9.9109e-25	2.7405e-19	2.7794e-36
NaN	NaN	NaN	4.5939e-01	7.8492e-03	1.4847e-03
NaN	NaN	NaN	NaN	6.6556e-02	5.6950e-05
NaN	NaN	NaN	NaN	NaN	1.1523e-08

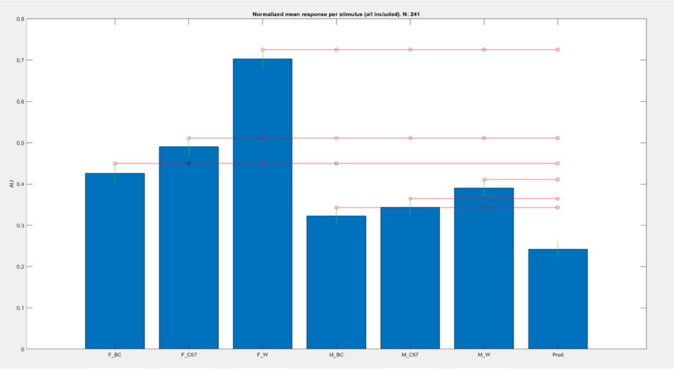

Supplement: Supplementary file 2 — Additional file 2. PDF file with details of statistical comparisons of responses for different categories. [file 12915_2021_1064_MOESM2_ESM.pdf]
