# Supplementary material for: Do all mice smell the same? Chemosensory cues from inbred and wild mouse strains elicit stereotypic sensory representations in the accessory olfactory bulb
Source: BMC Biol. 2021 Jun 28;19:133. doi: 10.1186/s12915-021-01064-7 (PMC8240315; doi:10.1186/s12915-021-01064-7)

# Additional Figure 3

## Population level distances for each strain

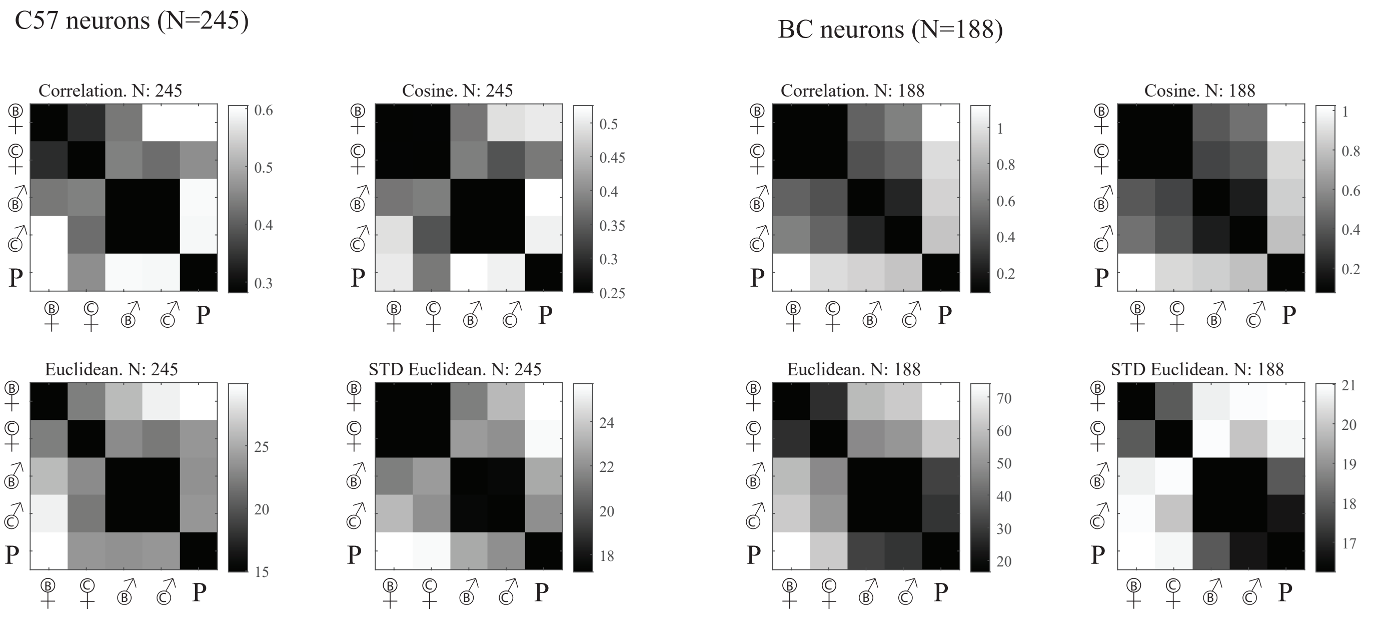


## Comparison of population level distances across the strains for 3 stimulus sets and 4 distance metrics

### population level distance (stim set 1)

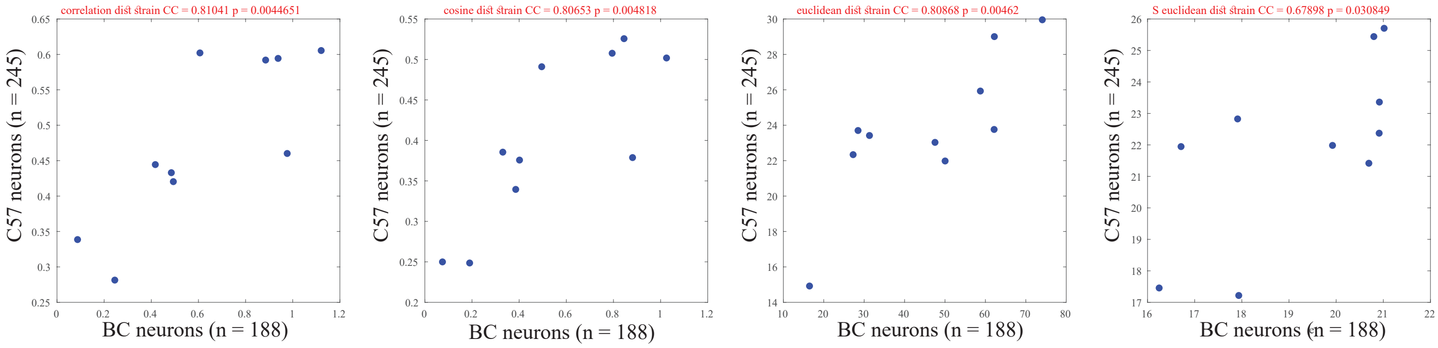


### population level distance (stim set 2)

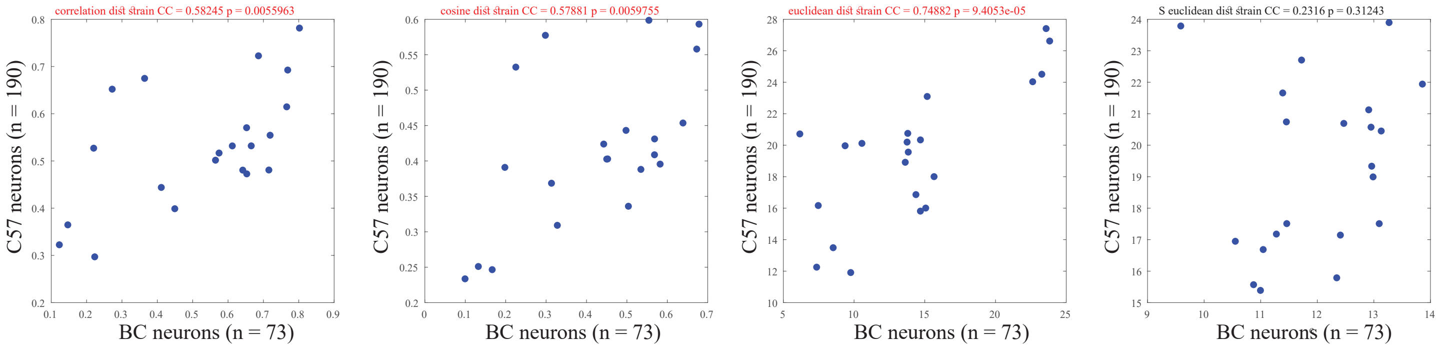


### population level distance (stim set 3)

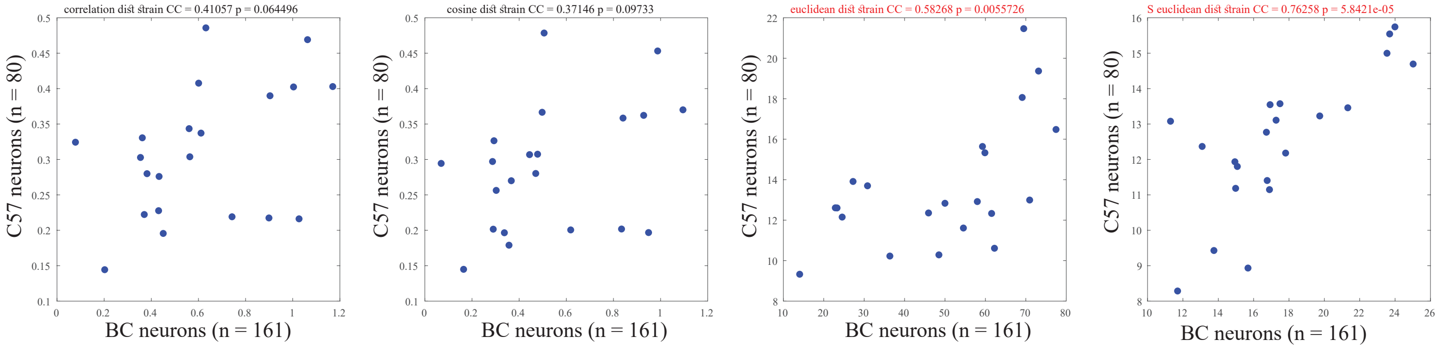

Supplement: Supplementary file 3 — Additional file 3. PDF file with similarity in responses across the two strains using various distance metrics. [file 12915_2021_1064_MOESM3_ESM.pdf]
