# Supplementary material for: Do all mice smell the same? Chemosensory cues from inbred and wild mouse strains elicit stereotypic sensory representations in the accessory olfactory bulb
Source: BMC Biol. 2021 Jun 28;19:133. doi: 10.1186/s12915-021-01064-7 (PMC8240315; doi:10.1186/s12915-021-01064-7)
Supplement: Supplementary file 4 — Additional file 4. PDF file showing bootstrap analysis to test the effect of sample size on the observed correlation in representations between the two strains (Fig. 4). [file 12915_2021_1064_MOESM4_ESM.pdf]

Additional file 4

Bootstrap analysis to test the effect of sample size on the observed correlation in representations between the two strains (Figure 4, panels B-D). Specifically, we repeated the analysis on set 1 (Figure 4B), with sample sizes equal to those available in datasets 2 (Figure 4C) and 3 (Figure 4D). Specifically, we repeatedly sampled a subset of neurons out of the total number available in stimulus set 1, and then recalculated the correlation based on the smaller sample. We did this for the sample sizes available in sets 2 and 3, 1000 times for each those sample sizes (in each of the 1000 iterations a different subset of neurons tested with stimulus set 1 was taken), for each of the 4 distances metrics (including those presented only in Additional File 3). The results are shown below. When 190 C57 neurons, and 73 BC neurons are taken, and when we consider the correlation distance measure (the same metric used in Figure 4), we see that the mean correlation drops to 0.7 (from 0.81 initially), while only in about 75% of the cases are the correlations significant at the 5% level. This is clearly above the 5% expected under the null hypothesis of no correlation, but implies that with a smaller sample size, there is indeed a realistic chance to underestimate the correlation and its significance. The correlations values and the frequency of significant correlations is even lower when using the sample size of stimulus set 3 (80 and 161 neurons). This analyses supports (but definitely does not prove) the statement that one potential reason for the smaller correlation observed in stimulus set 3 is the smaller sample size.

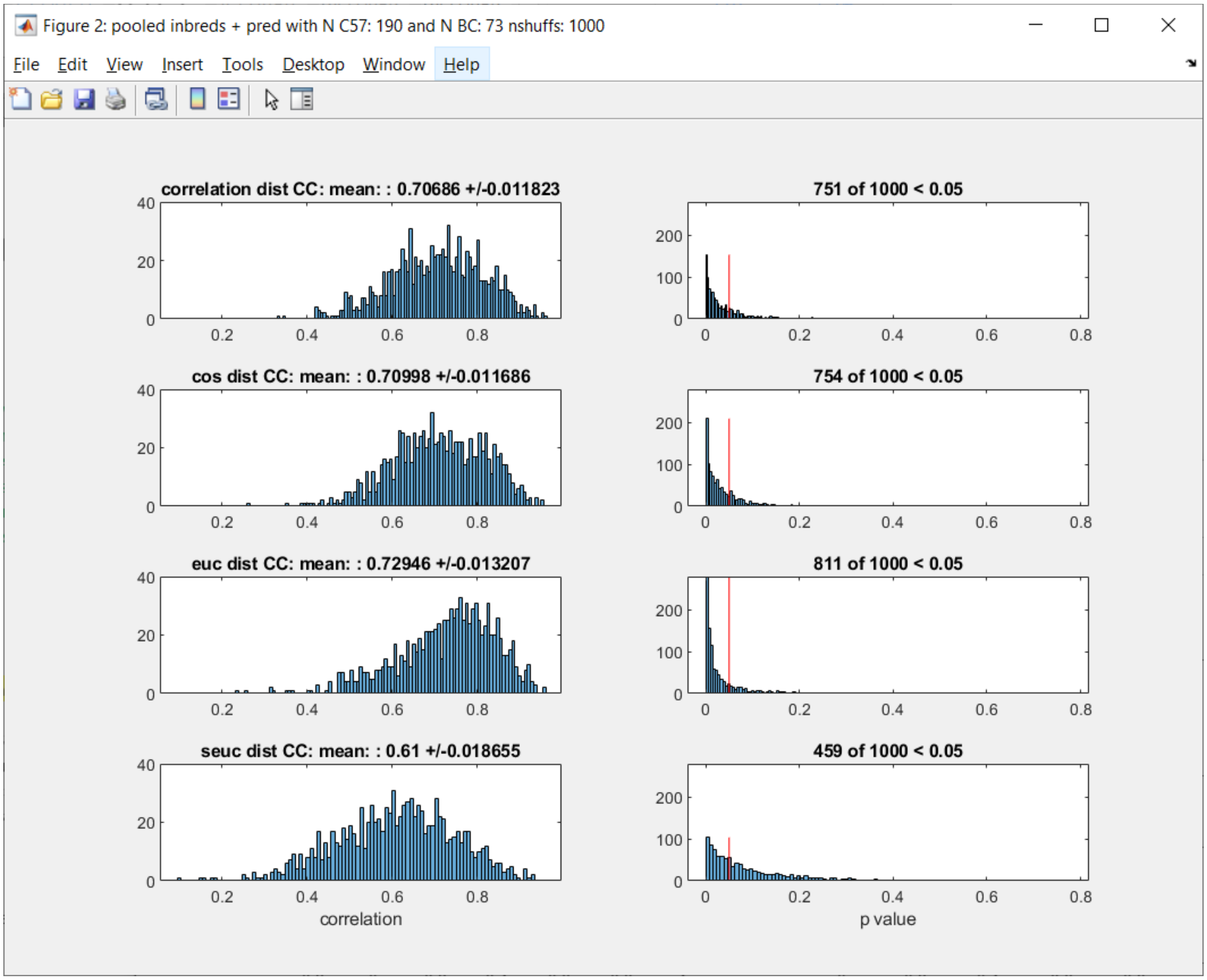
Figure 2: pooled inbreds + pred with N C57: 190 and N BC: 73 nshuffs: 1000
File Edit View Insert Tools Desktop Window Help
correlation dist CC: mean: : 0.70686 +/-0.011823
751 of 1000 < 0.05
cos dist CC: mean: : 0.70998 +/-0.011686
754 of 1000 < 0.05
euc dist CC: mean: : 0.72946 +/-0.013207
811 of 1000 < 0.05
seuc dist CC: mean: : 0.61 +/-0.018655
459 of 1000 < 0.05
correlation
p value

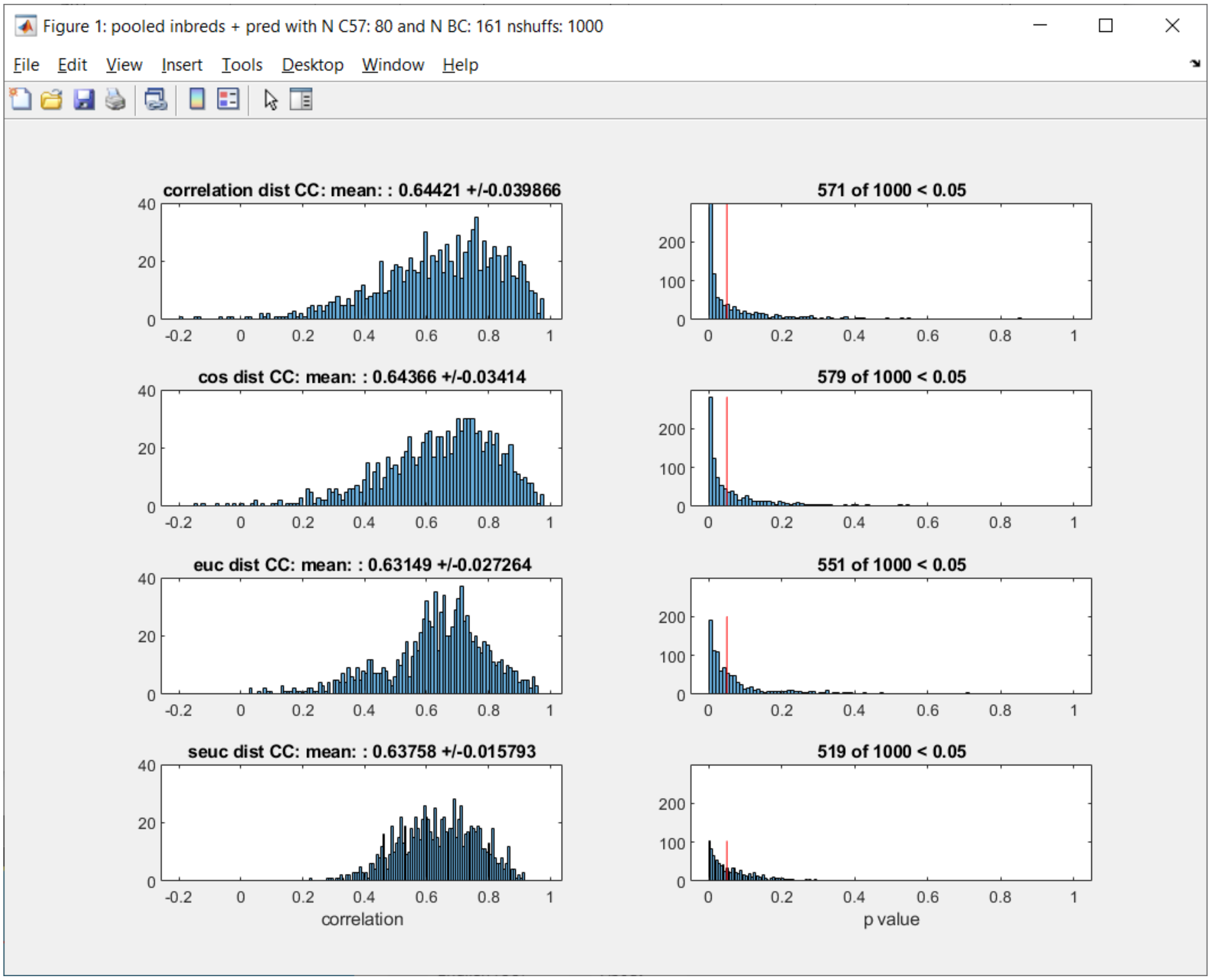
Figure 1: pooled inbreds + pred with N C57: 80 and N BC: 161 nshuffs: 1000
File Edit View Insert Tools Desktop Window Help
correlation dist CC: mean: : 0.64421 +/-0.039866
571 of 1000 < 0.05
cos dist CC: mean: : 0.64366 +/-0.03414
579 of 1000 < 0.05
euc dist CC: mean: : 0.63149 +/-0.027264
551 of 1000 < 0.05
seuc dist CC: mean: : 0.63758 +/-0.015793
519 of 1000 < 0.05
correlation
p value
