# Supplementary material for: Do all mice smell the same? Chemosensory cues from inbred and wild mouse strains elicit stereotypic sensory representations in the accessory olfactory bulb
Source: BMC Biol. 2021 Jun 28;19:133. doi: 10.1186/s12915-021-01064-7 (PMC8240315; doi:10.1186/s12915-021-01064-7)
Supplement: Supplementary file 5 — Additional file 5. PDF file showing a comparison of basic response features to the inbred stimuli from two different sources. [file 12915_2021_1064_MOESM5_ESM.pdf]

Additional File 5

Comparison of basic response features to the inbred stimuli from two different sources. The conclusion from these analyses that while responses are not identical, they are quite similar across the two datasets, despite the different source (and specific sub-strain) of the stimuli.

In these plots, stimulus name are as follows:
F_BC_PS: female BALB/cAnNCr purchased from Pathogen-free Facility of the Institute of Molecular Genetics (Czech Academy of Sciences in Prague). Stimulus set 3.
F_C57_PS: female C57BL/6NCrl purchased from Pathogen-free Facility of the Institute of Molecular Genetics (Czech Academy of Sciences in Prague). Stimulus set 3.
M_BC_PS: Male BALB/cAnNCr purchased from Pathogen-free Facility of the Institute of Molecular Genetics (Czech Academy of Sciences in Prague). Stimulus set 3.
M_C57_PS: Male C57BL/6NCrl purchased from Pathogen-free Facility of the Institute of Molecular Genetics (Czech Academy of Sciences in Prague). Stimulus set 3.

F_BC_HU: female BALB/C OLAHSD purchased from Harlan Laboratories (Jerusalem). Stimulus sets 1 & 2.
F_C57_HU: female C57Bl/6JRCCHSD purchased from Harlan Laboratories (Jerusalem). Stimulus sets 1 & 2.
M_BC_HU: Male BALB/C OLAHSD purchased from Harlan Laboratories (Jerusalem). Stimulus sets 1 & 2.
M_C57_HU: Male C57Bl/6JRCCHSD purchased from Harlan Laboratories (Jerusalem). Stimulus sets 1 & 2.

Thus, left side panels show the results for stimuli from dataset 3, while those on the right show results from stimuli from datasets 1 and 2.

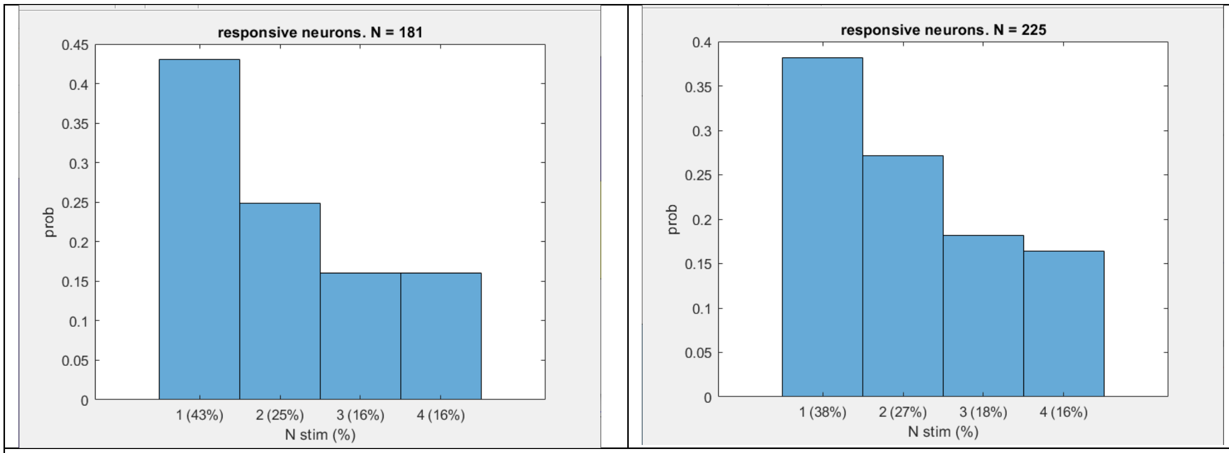

Fraction of neurons responding to 1,2,3, or 4 stimuli in each dataset. (neurons not responding to any of these stimuli are not included in these histograms).

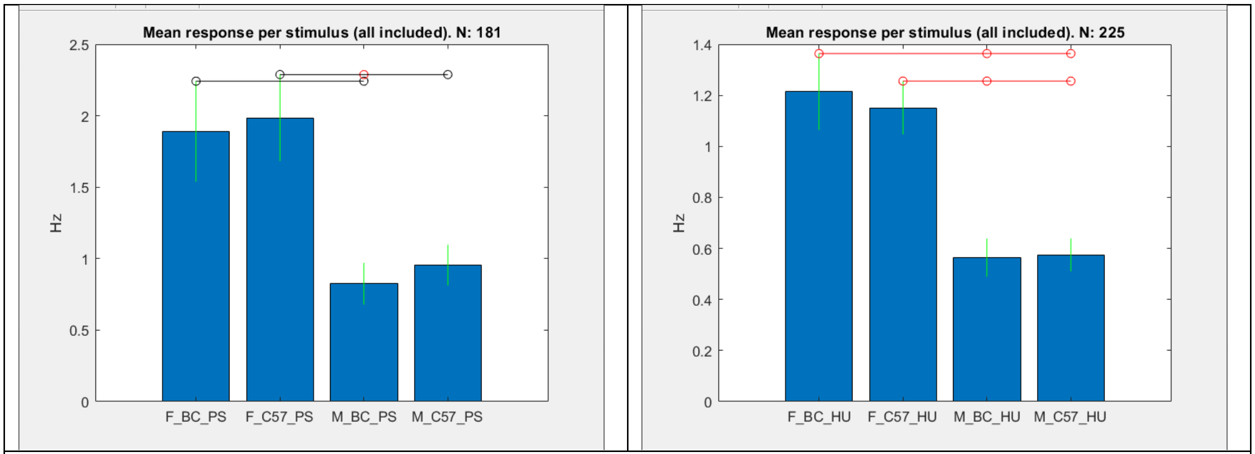

Mean response magnitude for all recorded neurons in each dataset. Vertical green error bars represent standard errors of the mean. Horizontal lines represent significant differences among stimulus pairs using a one-way non-parametric ANOVA (Kruskal Wallis test). Stimuli connected with lines with black circles indicate p values below 0.05, while red lines indicate p values below 0.01.

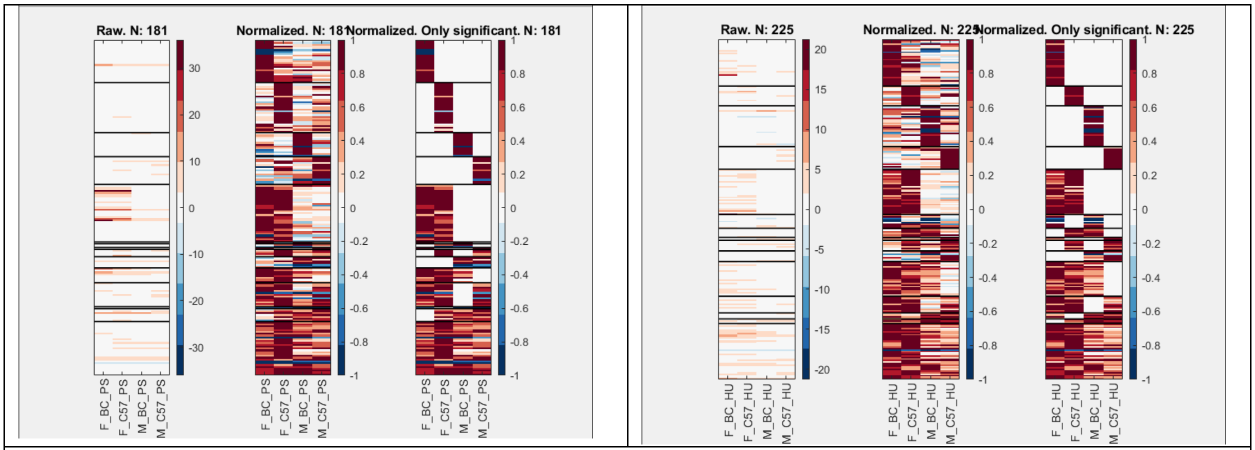

Raw (left), normalized (middle) and normalized and significant (right) response matrices for the entire dataset of neurons tested with each stimulus set.

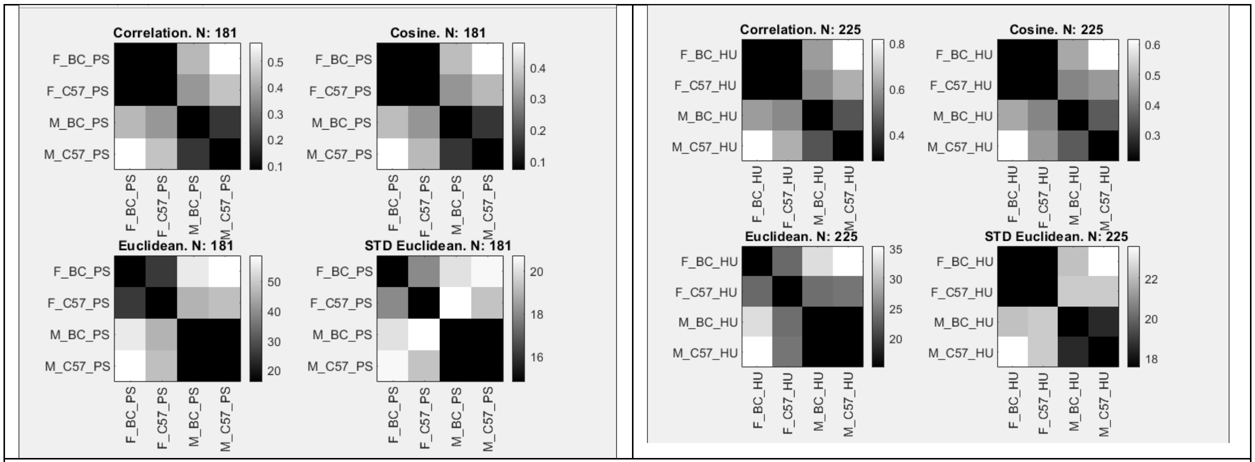

Distance matrices (using 4 different metrics) for the population response vectors for each of the 4 stimuli in each data set.

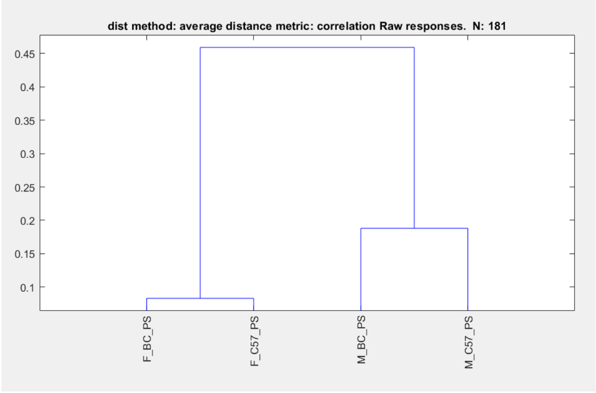

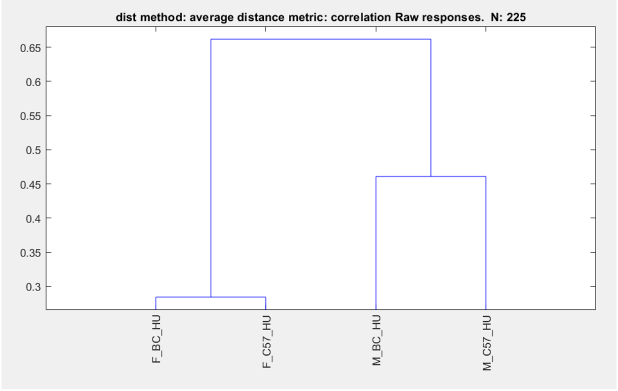

Hierarchical clustering based on correlation distance for population responses to each of the stimuli in the two data sets.
